# The magmatic system under Hunga volcano before and after the 15 January 2022 eruption

**DOI:** 10.1126/sciadv.adh3156

**Published:** 2023-12-15

**Authors:** Hélène Le Mével, Craig A. Miller, Marta Ribó, Shane Cronin, Taaniela Kula

**Affiliations:** ^1^ Carnegie Institution for Science, Earth and Planets Laboratory, Washington, DC, USA.; ^2^ GNS Science, Wairakei Research Center, Taupo, New Zealand.; ^3^ Department of Environmental Science, Auckland University of Technology, Auckland, New Zealand.; ^4^ School of Environment, University of Auckland, Auckland, New Zealand.; ^5^ Geology Unit, Natural Resources Division, Ministry of Lands and Natural Resources, Nuku‘alofa, Tonga.

## Abstract

One of the largest explosive eruptions instrumentally recorded occurred at Hunga volcano on 15 January 2022. The magma plumbing system under this volcano is unexplored because of inherent difficulties caused by its submarine setting. We use marine gravity data derived from satellite altimetry combined with multibeam bathymetry to model the architecture and dynamics of the magmatic system before and after the January 2022 eruption. We provide geophysical evidence for substantial high–melt content magma accumulation in three reservoirs at shallow depths (2 to 10 kilometers) under the volcano. We estimate that less than ~30% of the existing magma was evacuated by the main eruptive phases, enough to trigger caldera collapse. The eruption and caldera collapse reorganized magma storage, resulting in an increased connectivity between the two spatially distinct reservoirs. Modeling global satellite altimetry–derived gravity data at undersea volcanoes offer a promising reconnaissance tool to probe the subsurface for eruptible magma.

## Teaser

Satellite gravity data images the magmatic system under Hunga volcano and how it changed after the 2022 eruption.

## INTRODUCTION

**Fig. 1.**
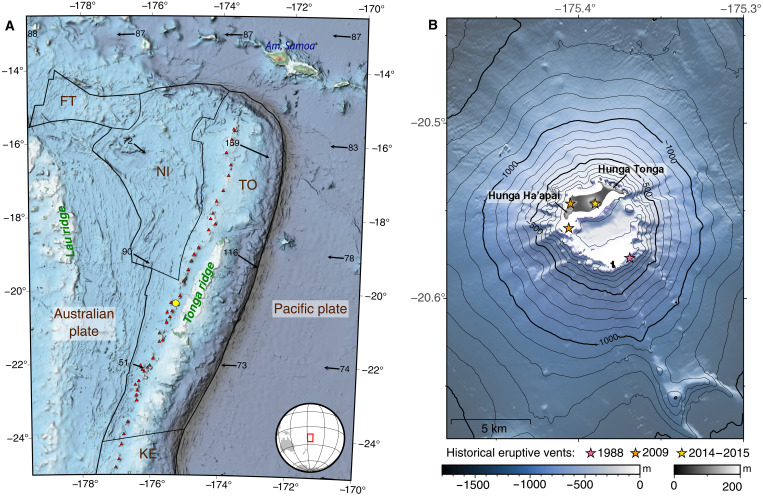
Map of Hunga volcano. (**A**) Tectonic setting of the Tofua volcanic arc, part of the Kermadec-Tonga subduction zone. Main volcanoes (red triangles), Hunga volcano (yellow circle), plate boundaries (black lines), and velocities relative to the Australian plate (black arrows, millimeters per year) from the MORVEL model (*67*). FT, Futuna; NI, Niuafo’ou; TO, Tonga; KE, Kermadec microplates. (**B**) Pre-2022 multibeam bathymetry of Hunga volcano and topography of the land extent as of September 2017 from (*68*).

On 15 January 2022, the largest explosive eruption recorded since Pinatubo in 1991 (*1*, *2*) occurred at Hunga volcano in the Tonga-Kermadec island arc (Fig. 1). In historic times, the energy released by the blast only compares to the VEI 6 1883 Krakatoa eruption (*3*). The Hunga eruption produced atmospheric and acoustic waves that propagated around the globe multiple times (*3*–*6*) and created large ionospheric perturbations (*7*), a 500-km-radius umbrella cloud extending as high as 57 km into the mesosphere (*8*–*10*), and an unprecedented amount of volcanic lightning (*5*). The acoustic-gravity waves from the explosion generated a global tsunami (*11*, *12*). The eruption had considerable repercussions across the Kingdom of Tonga islands due to tsunami impacts, ashfall, and the destruction of underwater communication cables. While the SO_2_ emissions were relatively low for an eruption of this magnitude (*13*) precluding global cooling, the unusually large amount of water injected into the stratosphere could be climate forcing and induce surface warming (*14*).

**Fig. 2.**
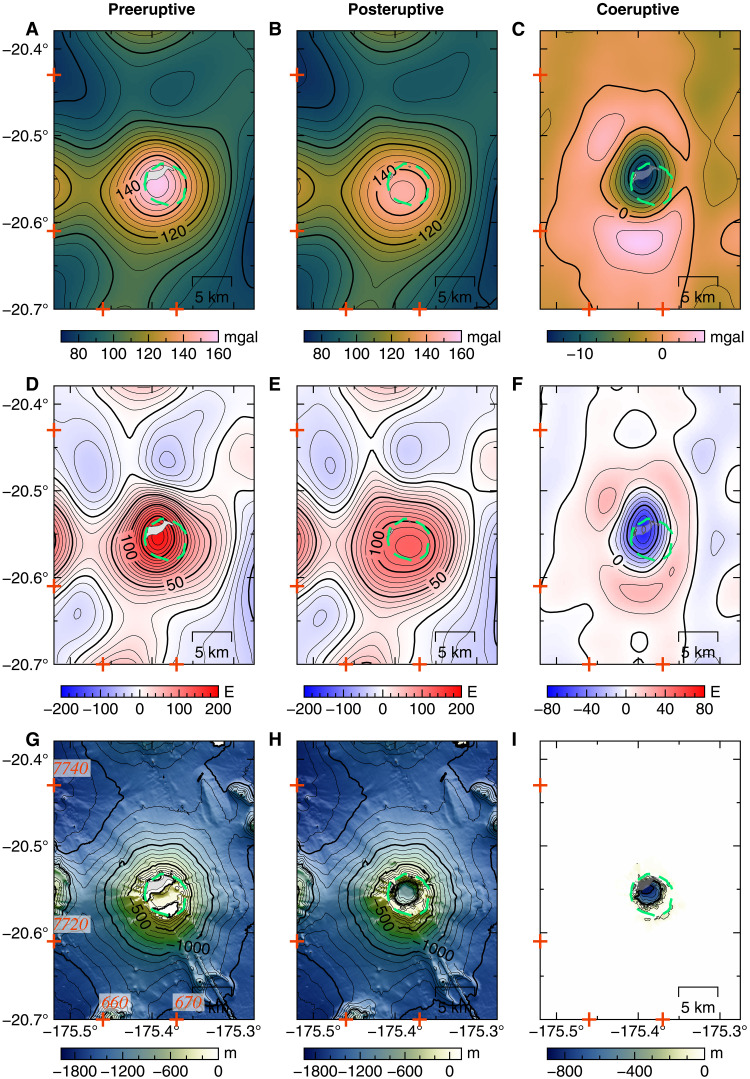
Pre-, post-, and coeruptive datasets. (**A** and **B**) Marine gravity anomaly derived from satellite altimetry [V31.1 and V32.1, respectively; (*21*, *23*)]. Subaerial island outlines in gray. (**C**) Change in marine gravity anomaly between (A) and (B). (**D** and **E**) Vertical gravity gradient (VGG) data from satellite altimetry [V31.1 and V.32, respectively; (*21*, *23*)]. (**F**) Change in VGG between (D) and (E). (**G**) Preeruptive bathymetry, (**H**) Posteruptive bathymetry (see Materials and Methods), and (**I**) change in bathymetry between (G) and (H) attributed to the January 2022 Hunga eruption. Caldera outline (green dashed line) and Universal Transverse Mercator (UTM) coordinates (zone 1) tick marks (in kilometers) shown for reference in red.

The two subaerial islands Hunga Tonga and Hunga-Ha’apai are the surface expression of a larger ~2-km-high volcano edifice with a ~5-km-diameter summit caldera (Fig. 1B). The paroxysmal 15 January 2022 eruption destroyed a cone that connected the islands since 2015 and reduced them to two thin slivers of land (Fig. 2H) (*15*). Historical eruptive activity at Hunga volcano has been Surtseyan in style in 2009 (*16*) and 2014–2015 (*17*) and confined to the caldera margins (*18*) (Fig. 1B). The climactic January 2022 eruption came after a 7-year hiatus in activity and a month-long unrest period, which started on 19 December 2021 and produced two explosive eruptions with ash plumes spreading to the tropopause (*10*). Repeat high-resolution multibeam bathymetry surveys show that the caldera floor collapsed by ~600 to 850 m following the 2022 eruption with >8 km^3^ of material being removed (*15*).

The well-documented, large magnitude and global effects of the 2022 Hunga eruption contrast with our lack of detailed information about the volcano subsurface structure and the sparsity of geophysical data available to study its magma plumbing system. Two key questions are raised by this eruption: Where and how much magma is stored in the crust under Hunga volcano, and how did the magmatic system change syn- and post-eruption? The amount of eruptible material present in a magma chamber is a key parameter controlling the size and timing of a future eruption (*19*, *20*). To understand the physical processes governing the 2022 Hunga eruption, we need to study the magma storage system and assess the state of the magmatic system before and after the eruption. Here, we use satellite altimetry–derived marine gravity data to provide constraints on the architecture of the magmatic system under Hunga volcano and explore how the exceptional 2022 eruption changed the magmatic system. Our findings have important implications for the assessment of volcanic hazards in the region associated with Hunga volcano. More generally, these results show that marine gravity data derived from satellite-altimetry together with multibeam bathymetry and geophysical inversion methods have the potential to uncover some key characteristics of magma storage at other understudied active submarine systems.

### Approach: Modeling vertical gravity gradient anomalies

Marine gravity anomalies derived from satellite altimetry have been used successfully in numerous studies of the ocean seafloor, its tectonic fabric, and seamount detection (*21*, *22*). Free-air marine gravity anomalies arise due to the gravitational attraction of the seafloor bathymetry and density variations within the crust and/or mantle; their spatial wavelengths reflect the depth of the anomalous source. Here, we are focusing on anomalies of kilometer-scale wavelengths reflecting structures in the oceanic crust. Marine gravity grids derived from satellite altimetry from (*21*, *23*) (V23.1 to 32.1, Materials and Methods) are used, and yearly changes in the gravity anomaly are calculated from 2014 to 2022. No significant change is observed from 2014 to 2021, before the August 2021 to August 2022 time interval (fig. S1 and Materials and Methods), which is the focus of this paper. To isolate the anomalies due to underground density variations, we model and remove the gravity effect of the bathymetry interface using two independent high-resolution multibeam bathymetry datasets acquired before and after the eruption, in 2016 and May 2022, respectively (Fig. 2, G to I, and Materials and Methods). The resulting Bouguer gravity anomaly is calculated for a range of crustal densities. We choose the reference density ρ_ref_ of 2750 kg/m^3^ as it minimizes the correlation between the Bouguer anomaly and the bathymetry grid (fig. S2).

We assume that all gravity changes that happened between August 2021 (V31.1) and August 2022 (V32.1) are due to the eruptive sequence that started in December 2021 and calculate the coeruptive gravity changes by subtracting the free-air gravity from the pre- and posteruptive time intervals (Fig. 2C). We then model the pre-, co-, and posteruptive residual Bouguer anomaly grids using a gradient-based mixed-norm inversion method (*24*) to determine the subsurface density distribution and its temporal evolution. We choose to use the vertical gravity gradient (VGG) anomalies (Fig. 2, D and E) as input to our models as gravity gradients are more sensitive to shorter wavelengths/shallow anomalous masses likely associated with magma reservoirs. Moreover, inversions of synthetic VGG gravity data for a shallow buried source indicate increased accuracy in the recovery of the source size and depth (figs. S3 to S6). By modeling gravity anomalies, we can interpret the resulting density contrasts to quantify parameters important for addressing our key questions: the location, depth, volume, and melt proportion of magma in the crust beneath Hunga volcano (*25*, *26*).

## RESULTS

### Preeruptive magmatic system

**Fig. 3.**
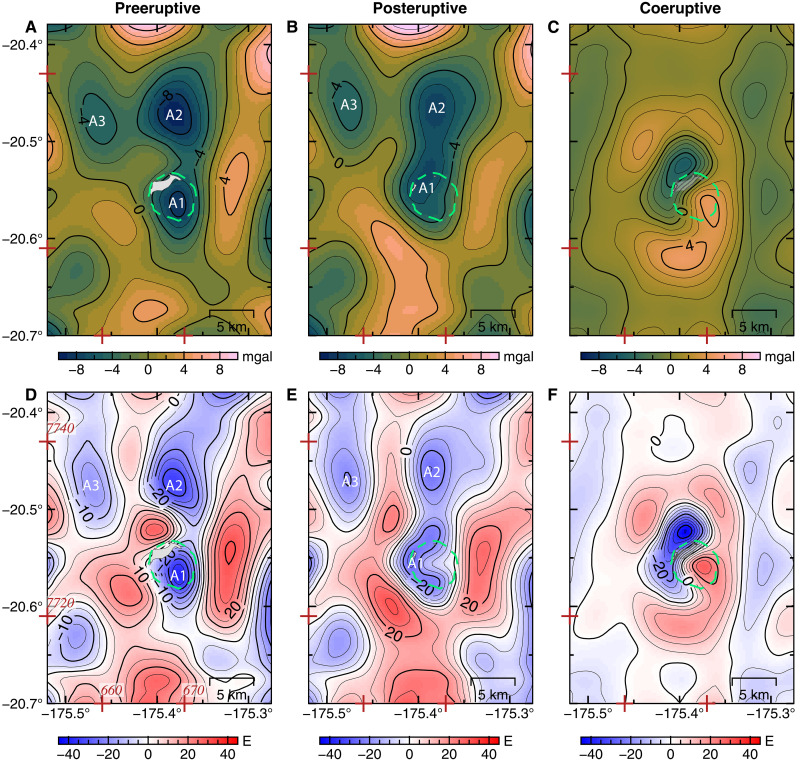
Pre-, post-, and coeruptive Bouguer gravity anomalies. (**A**) Residual Bouguer gravity anomaly calculated with a crustal density of 2750 kg/m^3^ and sea water density of 1027 kg/m^3^, after removing a quadratic polynomial regional trend. (**B**) Same as (A) but using posteruptive bathymetry and V32.1 of marine gravity anomaly. (**C**) Change in residual Bouguer gravity anomaly. (**D** to **F**) Residual Bouguer gravity anomaly and anomaly change calculated from the VGG data shown in (D), (E), and (F), respectively, and modeled in this paper. Caldera outline (green dashed line) and UTM coordinates (zone 1) tick marks (in kilometers) shown for reference in red.

**Fig. 4.**
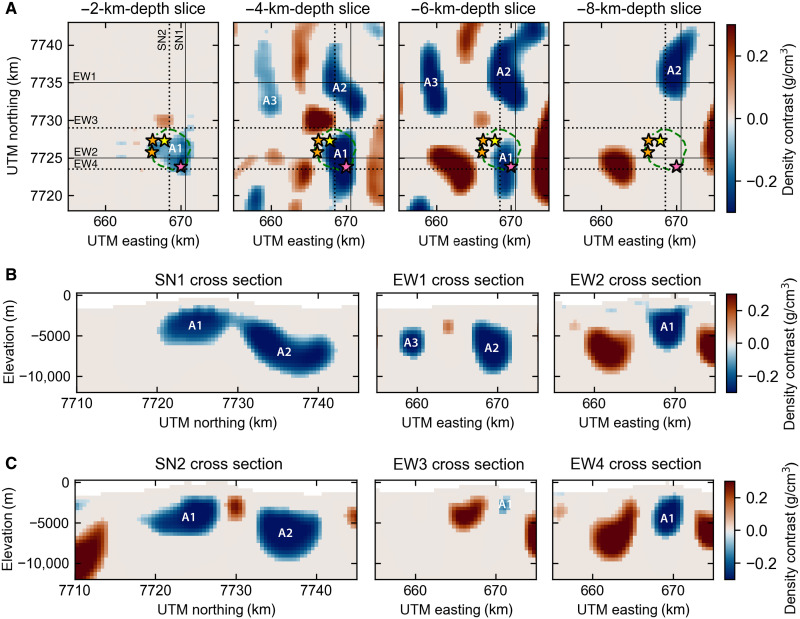
3D density model of the preeruptive magmatic system. (**A**) Depth slices and (**B** and **C**) cross sections through the density model. Inversion results shown for model norm [0, 2, 2, 2]. Model results for other tested norm combinations are shown in figs. S7 and S8. Color scale shows the density contrast with respect to a reference density of 2.75 g/cm^3^. Caldera outline (green dashed line) and historical vents (stars). Solid lines are optimal sections through the preeruptive low-density anomalies, and dotted lines are optimal sections through the posteruptive low-density anomalies, to be compared to the posteruptive density model of Fig. 5.

The residual Bouguer gravity anomaly shows gravity lows between −4 and −10 mgal [or residual Bouguer VGG between −15 and −40 Eotvos (E)] and highs up to 4 mgal (up to 30 E) (Fig. 3). Our three-dimensional (3D) density model (Fig. 4) reveals three low-density features, robust across the inversions using different combinations of model norms (figs. S7 and S8). First, a central low-density body underlies the caldera and the southern flank of Hunga (A1 hereinafter) and extends between 2- and 6-km depth below sea level (Fig. 4). Second, a larger, low-density body lies under the northern flank of Hunga (A2 hereinafter) and extends from ~3- to 10-km depth. Third, a smaller body to the northwest (NW) of Hunga (A3 hereinafter) extends from ~4- to 7-km depth. A1 and A2 are connected by a relatively narrow region at ~3-km depth with density contrasts between −0.05 and −0.1 g/cm^3^. The density contrast between the magma and the oceanic crust could be as low as −0.3 g/cm^3^, assuming an andesitic magma composition similar to the 2009 and 2014–2015 eruptions (see Materials and Methods).

### Posteruptive magmatic system

**Fig. 5.**
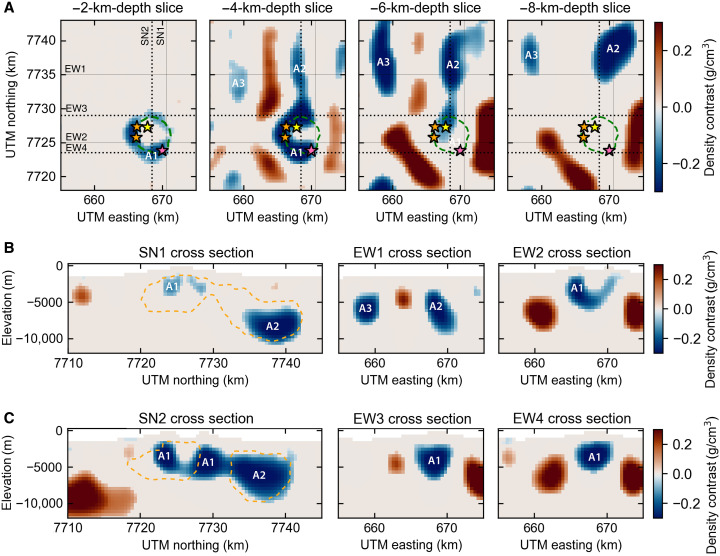
3D density model of the posteruptive magmatic system. (**A**) Depth slices and (**B** and **C**) cross sections through the density model. Inversion results shown for model norm [0, 2, 2, 2]. Model results for other tested norm combinations are shown in figs. S10 and S11. Color scale shows the density contrast with respect to a reference density of 2.75 g/cm^3^. Caldera outline (green dashed line) and historical vents (stars). Solid lines are optimal sections through the preeruptive low-density anomalies, and dotted lines are optimal sections through the posteruptive low-density anomalies, to be compared to the preeruptive density model of Fig. 4. Orange dashed line is the outline of the two main low-density anomalies for the preeruptive time interval shown on Fig. 4.

To study the effects of the 2022 eruption on the magmatic plumbing system at Hunga volcano, we use an updated marine gravity dataset (V32.1, Materials and Methods) compiled up to August 2022 and posteruptive bathymetry acquired in May 2022 to calculate and remove the gravity effect due to changes in seafloor topography caused by the caldera collapse. The difference in free-air anomaly shows a 12-mgal decrease over the August 2021 to August 2022 time interval (Fig. 2C). After correcting for topographic changes, the residual posteruptive Bouguer anomaly (Fig. 3, B and E) still shows three main negative density anomalies. The Bouguer anomaly changes are emphasized on Fig. 3 (C and F), and the corresponding 3D density model shows a large decrease in density (−0.6 g/cm^3^) centered north of the 2014–2015 eruptive vent and two areas of increased density around it (figs. S9 and S10). The model of the posteruptive Bouguer VGG anomaly shows that A2 and A3 remain mostly unchanged, while A1 displays a different ring-like pattern, at similar pre-2022 depths (2- to 6-km depth) (Fig. 5). The A1 low-density body now lies underneath most of the outer caldera rim. However, the area directly beneath the central part of the caldera has returned to a crustal density of ρ_ref_. Cross sections across our model results show that the connection between the northern low-density body (A2) and the central one (A1) is now ~2 km west of previously imaged and cover a wider area with lower density contrasts of ~−0.2 g/cm^3^, for all inversion scenarios (Fig. 5C and figs. S12 and S13).

### Crustal fluid volumes

**Fig. 6.**
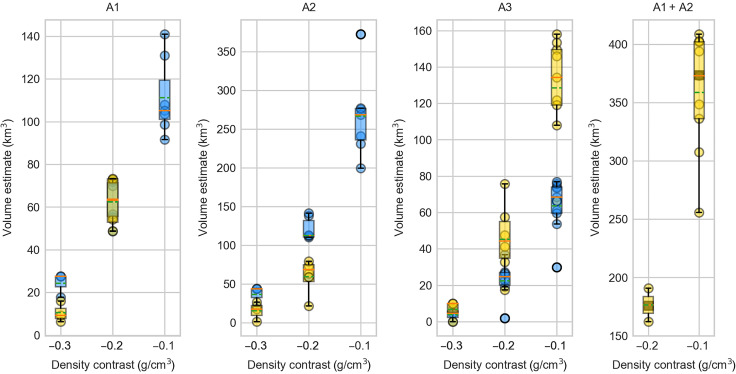
Volume estimates of the main modeled negative density bodies. Pre-2022 estimates (blue) and post-2022 estimates (yellow) presented for the inversion runs with three different model norm combinations ([1, 2, 2, 2], [1, 1, 1, 1], and [0, 2, 2, 2]) and three different isosurfaces for the central anomaly (A1), northern anomaly (A2), and northwestern anomaly (A3). A1 + A2 represents the volume of the two connected anomalies, after eruption. Green dashed lines in the box plots indicate the samples median, and orange lines indicate the mean. Symbols not contained in the box plots are outliers. Gray squares on the last panel are the sum of the preeruptive median volume values of A1 and A2 anomalies from the three model norms.

We then estimate the crustal volume affected by the low-density values as they reflect the potential presence of fluids (magmatic melt and volatiles and hydrothermal fluids/brines) in the crust. For all three negative anomalies, we calculate the volume enclosed by three isosurfaces of constant density contrasts, relative to the reference density: −0.1, −0.2, and −0.3 g/cm^3^ (Fig. 6). The estimated density contrasts are maximum/minimum values based on the chosen upper/lower bounds (±0.3 g/cm^3^) set for each inversion (Materials and Methods). The larger the imposed density contrast, the smaller the body needs to be to explain the amplitude of the observed gravity anomaly. During the preeruptive time interval, for the A1 low-density body, we find median volumes of 27 [95% confidence interval (CI) [23, 31]], 63 (95% CI [53, 73]), and 105 (95% CI [94, 116]) km^3^ for the density contrasts of −0.3, −0.2, and −0.1 g/cm^3^, respectively. Similarly, A2 volume ranges from 43 (95% CI [33, 54]) to 268 (95% CI [244, 292]) km^3^ and A3 from 5 (95% CI [1, 8]) to 68 (95% CI [61, 76]) km^3^ (Fig. 6). Other regions imaged NW and southwest (SW) of the caldera have densities higher than the reference density (density contrasts of 0.2 to 0.3 g/cm^3^; Fig. 4).

During the posteruptive time interval, the volume estimates for the core of the anomalies A1 and A2 (−0.3 g/cm^3^) have decreased by 66 and 59%, respectively, after the eruption (Fig. 6), indicating mass loss. On the other hand, the regions with smaller density contrasts (−0.1 and −0.2 g/cm^3^, A1 + A2 combined anomaly, Fig. 6) did not change in volume significantly before and after eruption. These estimates illustrate the substantial reorganization of density specifically below the summit caldera.

To summarize, before the 2022 eruption, there is evidence for three regions of significant low density directly under, north and northwest of the caldera, that we interpret as reflecting the Hunga magmatic system. The eruption caused substantial changes in the magmatic system including anomalously low-density regions around the caldera rim, regions with increased density, and a new low-density connection between the two main reservoirs (A1 and A2). Next, we discuss the interpretation of these density contrast models in terms of magma system architecture and dynamics.

## DISCUSSION

### Interpretation of gravity anomalies and limitations

There are three possible interpretations for a low-density region in the upper oceanic crust: the presence of magma (magmatic melt and volatiles), hydrothermal fluids, or sediments. A sediment infill of a topographic low, for example, a caldera, would create a negative gravity anomaly with a much shorter wavelength due to its surficial nature and larger magnitude and display a double polarity VGG (positive/negative VGG), which are not consistent with our observations. We therefore discard this hypothesis. The lava compositions of the last two eruptions at Hunga in 2009 and 2014–2015 were typical of basaltic andesite (*27*). The 2022 deposits also show an andesitic composition with whole rock SiO_2_ 57 wt % (*28*). The estimated density of melt with an average 2009 and 2014–2015 whole rock composition varies between 2.4 and 2.6 g/cm^3^ (Materials and Methods). The presence of andesitic melt in the crust would then create a density contrast from −0.35 to −0.15 g/cm^3^ with respect to our reference density ρ_ref_. Therefore, assuming the lowest possible melt density (2.4 g/cm^3^) and no other fluids are present, the density contrast isosurface of −0.1 g/cm^3^ could represent a crustal region containing up to 28% volume fraction of melt, the −0.2 g/cm^3^ up to 57%, and −0.3 g/cm^3^ up to 85%.

Hydrothermal fluids form when seawater interacts with the heated crust, due to the presence of a magma chamber, and thus active hydrothermal systems are often found at submarine volcanoes. Their presence is evidenced by hydrothermal manifestations on the seafloor. Active hydrothermal systems have been reported and studied at submarine arc volcanoes, e.g., at Brothers volcano (*29*, *30*) and Clark volcano (*31*) of the Kermadec arc. Hydrothermal fluids, depending on their composition, depth, and temperature have a much lower density than magmatic melt, from ~0.1 to ~1.0 g/cm^3^, and fill available pore space in the rocks above the main magma reservoir. Thus, hydrothermal systems (recharge/discharge and reaction zones) are usually found at shallow depths and confined to the upper few kilometers of the crust (*30*, *32*). Lithic analysis of the tephra fall shows hydrothermally altered clasts (~5% of lithic component), providing evidence for a localized hydrothermal system.

Long-term magma storage in the crust typically happens around 5- to 7-km depth at arc volcanoes (*33*, *34*). Consequently, we interpret our low-density anomalies as regions with significant accumulation of magma (melt and crystal mush zone) and for the shallower depths (<4 km), likely a combination of magma and hydrothermal fluids. Similarly large volumes of stored magma have been estimated under active, subaerial volcanoes both based on geophysical surveys and geochemical analyses (*35*–*37*). For example, Weber *et al*. (*37*) estimate a magma volume of 350 to 400 km^3^ stored under Toluca volcano. Following the conceptual models proposed for other tectonic contexts—stacked sills for fast-spreading ridges (*38*) and the crystal-rich transcrustal magmatic system for continent and silicic melt storage (*39*)—we propose that each low-density body represents stacked magma lenses or sills that cannot be individually resolved by gravity. As a result, our geophysical models may overestimate melt volumes, as they average both magmatic sills and the solid crust between them. Only a portion of the total magma volume is eruptible, often defined as requiring more than 50% volume fraction of melt (*40*). As for the high-density anomalies, we suggest that they represent previously intruded melt lenses that have cooled and solidified, resulting in higher density (up to ~3.0 g/cm^3^). These plutonic rocks thus indicate that former reservoirs and feeding systems developed over the life span of the volcano.

Our interpretation is limited by the accuracy and resolution of the satellite altimetry–derived gravity data, which, in turn, are limited by the sea surface roughness from ocean waves, the number of datasets available from multiple altimeters, and the upward attenuation of the gravity field from the seafloor to the sea surface (*23*, *41*). Using the 1–arc min resolution marine gravity grid, only kilometer-scale wavelength features can be resolved. In this study, our temporal constraints are limited by the acquisition dates of the bathymetric surveys and the 1-year time interval between updated marine gravity models derived from satellite altimetry. We then assume that all gravity changes observed between August 2021 and August 2022 are coeruptive changes that happened during the entire eruptive sequence. Therefore, this analysis can only detect major changes in the magmatic system, producing gravity anomalies >2 mgal over a yearly time interval, corresponding to relatively large mass movements that would take place during major eruptions (*42*).

Thus, we lack a detailed picture of the architecture of the magmatic system and mass transfer between the different reservoirs during the eruptive sequence at Hunga volcano. For example, pathways between lenses of melt and to the surface may exist, but the induced gravity anomaly would be lower than our detectability limit. The acquisition of shipborne gravity or reflection seismic data with a tight line spacing, targeting the satellite-derived anomalies, would be a complementary dataset to help get a more detailed picture of the conduit system. Magmatic systems under other submarine volcanoes have been imaged using passive and active seismic methods. For example, seismic reflection data imaged the fine-scale melt distribution under Axial seamount (*43*, *44*), 3D velocity models from tomographic inversions revealed the architecture of the magmatic systems under the offshore Mayotte (*45*) and Santorini (*46*) volcanoes, and full waveform inversion detected a small magma chamber under Kolumbo submarine volcano offshore Santorini, Greece (*47*). While we assume that the gravity changes happening over the 2021–2022 interval are dominated by coeruptive changes, posteruptive gravity changes of smaller amplitude could arise from small residual fluid movement (melt or hydrothermal fluids re-equilibrating after eruption) or changes to the seafloor morphology (e.g., landslide and pyroclastic density current deposits) as a result of the eruption column and caldera collapse and magmatic system readjustments. Last, the gravity-derived density corresponds to a bulk magma density, and we cannot distinguish between the individual phases that make up the magma. When interpreting the bulk density estimates, we should keep in mind that we expect gradational boundaries within and around the reservoir with different proportions of each magma phase. The analysis of the 2022 erupted deposits shows a glass-rich and crystal-poor composition, as was observed for past large-magnitude eruptions at Hunga with a dominant crystal content <10 to 15% (*18*). Therefore, melt will be the dominant component, and volume estimates will represent maximum values as the presence of crystals will increase the density of magma, reducing the overall density contrast. While our gravity dataset cannot resolve the exact fraction of eruptible melt present in the imaged reservoirs, it brings essential broad constraints on the total volume of stored magma and the extent and depth of the magmatic reservoirs.

### Magmatic system dynamics from coeruptive surface and subsurface changes

Following the January 2022 eruption, a repeat high-resolution multibeam survey took place in May 2022 and revealed notable geomorphological changes at Hunga volcano. The central part of the caldera floor collapsed by ~600 to 850 m, and the edifice built in 2015 connecting the two islands was removed by the blast (Fig. 2H). Large erosional channels radiating out from the summit vents indicate that turbulent, underwater, and pyroclastic density flows traveled as far as 100 km, which could explain the destruction of underwater communication cables. The rest of the Hunga volcano edifice remains mostly unchanged (*15*). On the basis of deposit sampling, a total of ~9.5 km^3^ of material was ejected by the 2022 eruption, most of it deposited within 20 km of the caldera rim or ejected into the atmosphere. On the basis of differencing between the two bathymetric datasets before and after the eruption, ~8 km^3^ of volume loss can be accounted for. Because of magma compressibility due to the amount of exsolved gas in the chamber, we cannot directly equate the estimate of the magma chamber volume change at depth over an eruptive episode to the volume of erupted material; the latter is much greater (*48*).

**Fig. 7.**
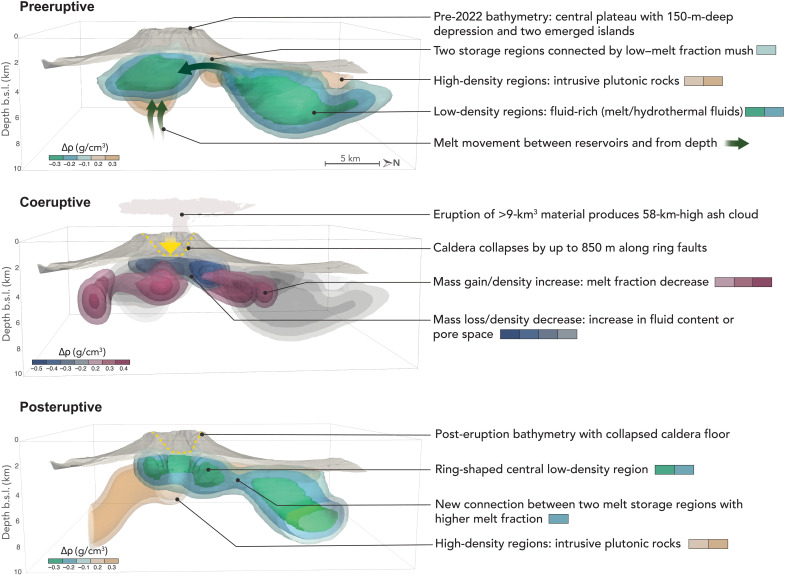
Interpretive model for the Hunga magmatic system evolution. Proposed architecture and dynamics of the Hunga magmatic system from the gravity models presented in this study. 3D volumes represent isosurfaces of constant density contrasts. Bathymetry (gray surface) is exaggerated vertically 2.4 times, and ash plume is not drawn to scale. Green arrows show the inferred movement of magmatic and/or hydrothermal fluids from one reservoir to another and replenishment from a deeper source. b.s.l., below sea level.

In the subsurface, the coeruptive density model shows several areas experiencing a mass/density increase or decrease (figs. S9 and S10). A variety of magmatic processes can explain these observed gravity changes including the movement of magma or hydrothermal fluids between reservoirs, a change in porosity, a change in melt fraction/crystal content, or syn-eruptive crystallization due to rapid decompression of the magma reservoir (fig. S11). The largest change in density happens slightly to the north of the 2014–2015 and 2022 eruptive vents with a change of −0.6 g/cm^3^ happening at ~4-km depth (Fig. 7 and figs. S9 and S10). The large density decrease of previously solid crustal rocks can be explained by magma recharge—from depth or from lateral magma movement—following the sudden removal of a large volume of magma from the main reservoir during the paroxysmal eruption (fig. S11). Alternatively, the observed gravity decrease could record the ring faults, and fractured rocks formed during caldera collapse, possibly filled with hydrothermal fluids; however, these are likely to produce lower amplitude gravity changes. The posteruptive density model shows a ring-shape area of low density (Fig. 5A). This area is similar in depth as the preeruptive central magma reservoir, but the lack of anomaly in the center indicates a return to the reference density. This central area could indicate the part of the reservoir tapped by the eruption. Underwater gas plumes extending up to 30 m above the seafloor were identified at the edges of the caldera during the May 2022 bathymetric survey (*49*). The most vigorous ones align in a ~north-south trend along the center of the caldera, indicating post-eruption shallow magma degassing. During a repeat survey in October 2022, the activity of these vents had subsided, and shallow hydrothermal vents remained on the NW inner caldera wall. These observations are consistent with our imaged magmatic system with a low-density melt- and volatile-rich magma body starting as shallow as 2-km depth under much of the caldera margins.

We hypothesize that the sudden and large evacuation of magma from the main reservoir (A1) caused the chamber roof (~2-km thick) to founder. As a result, the caldera collapse outlines the surface expression of the portion of the reservoir tapped during the eruption. Experimental results show that the critical erupted magma chamber volume fraction required to trigger caldera collapse depends mainly on the chamber roof aspect ratio (*50*). Here, for an aspect ratio between 0.5 and 0.8 (defined as magma chamber roof thickness over width), a critical volume fraction between 30 and 40% would be necessary to trigger collapse. Assuming the magma reservoir with the highest melt fraction is delineated by the isosurface of −0.3 g/cm^3^, the volume of stored melt would be 27 km^3^ (95% CI [23, 31] km^3^). Using the upper end-member estimate of 8 km^3^ for the volume of erupted material yields an erupted volume fraction of ~30%, consistent with experimental observations of caldera collapse. The northern and eastern extent of the central magma reservoir (A1) appears to be structurally controlled as it aligns with the caldera rim (Fig. 4A). The presence of steeply dipping ring faults can explain the sharp lateral changes in VGGs observed around the caldera (Fig. 3). The chamber roof aspect ratio found in this study (ratio < 1) puts the Hunga caldera collapse into the piston-like subsidence regime bounded by nearly vertical ring faults (*50*), similar to recent caldera collapse observed at Kilauea (*51*) and Bardarbunga (*52*, *53*) volcanoes.

### A multireservoir magmatic system

This study provides geophysical evidence for a complex, multireservoir system at Hunga volcano. The northern low-density body (A2) extends down to 10 km and could correspond to a secondary magma reservoir. This reservoir is offset ~5 km to the north compared to the caldera summit and its underlying reservoir (A1). At arc volcanoes, magma reservoirs are often offset from the main volcanic edifice (*54*–*56*), and lateral magma flow, sometimes over great distances, has been observed frequently at basaltic systems and more rarely at rhyolitic caldera systems (*39*, *51*, *52*). A porous mush zone, adjacent to the main reservoir, was also proposed by (*57*) to explain the varying effusion rate during the long-lived 2018 basaltic eruption at Mayotte volcano. Here at Hunga volcano, we image a similar magmatic system architecture with a main reservoir centered under the caldera and a secondary offset reservoir/mush zone under the northern flank. Thermobarometry of the 2009 and 2014–2015 magmatic products from Hunga volcano indicates the presence of a single shallow storage reservoir at 5- to 8-km depth subject to convective mixing, homogenization, and steady-state supply of primitive basaltic andesite (*18*). These smaller-scale Surstseyan-style eruptions, similar to the eruptive activity observed in December 2021, would tap the shallower system (A1) as evidenced by the homogeneous andesite composition of the pyroclastic deposits (*18*). Preliminary isotopic measurements and magmatic volatiles analysis from the 2022 eruptive products indicate that different andesitic magma sources mingled both before and during the eruption (*28*, *58*, *59*). The large-scale caldera-forming January 2022 eruption, therefore, would have tapped several parts of the magmatic system (A1 and A2). Our finding of multiple, connected magma reservoirs underneath Hunga is consistent with this hypothesis. As the shallow central magma reservoir (A1) fed the eruption, the pressure would have dropped and allowed magma to flow from the northern reservoir (A2) and from depth, as illustrated in our interpretive model (Fig. 7). The high SO_2_ emissions recorded during the month-long unrest—significantly higher than before the 2009 and 2014–2015 events—provide an additional piece of evidence supporting the rejuvenation of the magmatic system before the paroxysmal event (*13*). The tapping of deeper parts of the magmatic system and possibly magma injection from a deeper, gas-rich source may also explain the violence of the 2022 eruption (*28*).

In our interpretive model of the Hunga magma plumbing system (Fig. 7), magma pathways between the two reservoirs could form shortly before or during an eruption or may exploit a preexisting feature. On the basis of the short timescales of magma mingling, in the order of minutes, derived from volatile diffusion studies, the formation or reactivation of these pathways could occur rapidly (*58*, *59*). One of the main changes observed in our 3D density model results after the eruption is the new and increased connection between the central and northern storage zones, as illustrated by the lower density contrast and wider footprint of the new anomaly between A1 and A2 (Fig. 5C). Our results provide strong evidence for a change in magmatic connectivity between the two main reservoirs (A1 and A2) following the 2022 eruption. Magma from the northern reservoir (A2) would have transferred to reservoir A1 through this newly established pathway. On the contrary, the third low-density region (anomaly A3) located to the northwest does not appear to connect to the other reservoirs, and we therefore assume that it represents an older, solidifying mush zone, no longer able to feed the A2 reservoir. We therefore hypothesize magma replenishment of reservoir A1 both from deeper sources, not resolved in our data, and from the adjacent A2 reservoir.

### Implications

This study provides a snapshot of the state of a multireservoir magma system before and after one of Earth’s largest witnessed eruptions. We show that the magmatic system under Hunga volcano is spatially complex with a large amount of magma with high melt content stored in two reservoirs. Even with as much as 66% of the stored magma evacuated or removed during the 2022 eruptive sequence, we estimate that two crustal fluid regions containing up to 85% volume fraction of melt remain. This corresponds to up to 9 (95% CI [5, 14]) and 17 (95% CI [6, 29]) km^3^, respectively, of remaining eruptible magma still stored in two reservoirs. Understanding the conditions and configuration of melt storage under Hunga—depth and volume—is crucial to better assess volcanic hazards in the Tonga archipelago and will eventually help provide a more accurate forecast of the size and likelihood of future eruptions. On the basis of the location and extent of magma reservoirs mapped in this study, future shipborne surveys to collect additional geophysical data should not solely focus over the central, summit caldera but extend on the flanks. This eruption also illustrates the potential risks posed by other unstudied submarine volcanoes. More than 75% of Earth’s volcanism happens under the oceans (*60*), and most submarine volcanoes lack the instrumentation needed for effective continuous monitoring. Rare geophysical observations come from very distal, island-based, networks. When independent, multibeam bathymetry datasets exist, modeling widely available satellite altimetry–derived gravity data, which covers most undersea volcanoes, offers a promising reconnaissance tool to probe the subsurface for melt. Our approach would thereby help identify understudied or new active submarine magmatic systems with the potential to produce similar scale eruption to the 15 January 2022 Hunga eruption.

## MATERIALS AND METHODS

### Gravity data and analysis

First, we use marine gravity anomaly grids V23.1, 24.1, V25.1, V27.1, V29.1, V30.1, V31.1, and V32.1 (*21*) and calculate the yearly change in anomaly over the study area from 2014 to 2022. The uncertainty shown on fig. S1 (pink area) is the quadratic sum of each year’s maximum uncertainty. Overall, the uncertainty decreases with time from >6 mgal in 2014 to <4 mgal in 2022 as more data are acquired. No gravity anomaly change greater than the uncertainty is observed from 2014 to 2021.

For the 2021 to 2022 time period, we use global satellite altimetry–derived marine gravity anomaly and VGG grids V31.1 (08/2021) and V32.1 (08/2022) for the pre- and posteruptive time period, respectively (*21*, *61*–*63*). The compilations include gravity data derived from the following altimeters: Altika, Cryosat LRM, Cryosat SAR, Sentinel-3A/B, Jason-2, and Cryosat-2 (*23*). The mean uncertainty of the marine gravity anomaly, both for the pre- and posteruptive time intervals, is less than 2 mgal over our study area (figs. S14 and S15). The gravity effect of the bathymetry interface is computed using Parker’s expansion (*64*) up to the fourth terms, as implemented in gravfft in Generic Mapping Tools (*65*). The Bouguer anomaly is calculated for crustal densities varying from 2200 to 2900 kg/m^3^. We assume that the best correction density is the one that minimizes the correlation of the Bouguer anomaly and topography. Least-square slope estimates (L2 slope on fig. S2) and Pearson correlation coefficient (*r* on fig. S2) between the Bouguer anomaly and the bathymetry are both minimized for a correction density of 2750 kg/m^3^, which is chosen as the reference density for the rest of the study (fig. S2). We obtain the residual Bouguer anomaly by removing a long wavelength regional field estimated using a quadratic polynomial (degree 2, six terms). The quadratic polynomial is preferred over degree 0 and 1 polynomials that oversimplify the regional field and do not model the curvature of the field, and over a bicubic, degree 3, polynomial that is more complex and could introduce artifacts (figs. S16 and S17).

### Bathymetry datasets

Bathymetry datasets are independent from the satellite-derived gravity data and acquired from multibeam echosounders. The preeruptive bathymetry data are a multibeam bathymetric mosaic from the National Centers for Environmental Information (NCEI) at the National Oceanic and Atmospheric Administration (NOAA) with 3–arc sec resolution. Most of the lines around Hunga volcano are from the R/V Falkor 2016 survey (FK160407) using a Kongsberg EM302 multibeam echosounder. The surveys within the caldera were carried out in November 2015 using a WASSP multibeam echosounder by S. Cronin on the vessel Pacific Rose. The posteruptive high-resolution multibeam bathymetric data were acquired in May 2022 during a research cruise led by S. Cronin on the Tongan Vessel Pacific Horizon, using a WASSP multibeam echosounder. The same WASSP instrumentation was used for a repeat survey led by S. Cronin and M. Ribo on the Tongan Vessel Pacific Dawn in October 2022. Before calculating the terrain correction, we apply a low-pass Gaussian filter (SD of 2 km and width of 12 km) to the bathymetry to filter out small wavelength content that is not present in the gravity data due to upward attenuation of the gravity field from the seafloor to the sea surface (fig. S18). The bathymetry grids are then downsampled to 1 arc min to match the gravity data sampling resolution.

### Gravity data inversion

Both the forward simulations and inversions are implemented with the SimPEG (*24*) open-source package. The composite objective function contains the data misfit and model regularization terms in a Tikhonov framework. The inverse problem is then solved using a mixed 𝓁*p*,*q* norms regularization with *p*,*q* ∈ [0, 2] generating more compact or smooth models (*66*) and is optimized iteratively using a gradient-based Gauss-Newton algorithm. Depth weighting is applied in SimPEG to counteract the natural (1*/z*^2^) decay of the gravity kernel function. We discretize the model space using an octree mesh of base cubic cell size of 450 m and 5-km padding on each side. The reference model is set to 0 g/cm^3^ everywhere, while the starting model has a density contrast of 1 × 10^−4^ g/cm^3^ over the entire domain. Residuals between observed and modeled VGG anomalies are shown on figs. S19 to S21. Histogram of residuals show a single or double distribution with the mean centered on 0 and residuals always smaller than 1.5 E. We show the 3D density models resulting from the inversion with model norm combination [0, 2, 2, 2] as it consistently yields the lowest misfit. To provide a range of magma volume estimates and calculate uncertainties, we use the inversion results from the three different norm combinations [1, 2, 2, 2], [1, 1, 1, 1], and [0, 2, 2, 2] that best recovered the source parameters in the synthetic test. To estimate the expected density of magma at depth, we conduct MELTS thermodynamic equilibrium simulations (isobaric) for the average oxide compositions of 2014–2015 and 2009 tephra samples from (*27*). We find densities between 2.4 and 2.58 g/cm^3^ at 108 MPa (~4-km depth) and between 2.42 and 2.6 g/cm^3^ at 216 MPa (~8-km depth), for a water content from 1 to 4 wt %, respectively, and melt temperatures 1000° to 1200°C. These values are consistent with preliminary thermobarometry results, indicating temperatures of 1110° to 1130°C and pressures of 150 to 200 MPa (*28*). On the basis of this estimate, we choose the lowest bounds for our pre- and posteruptive gravity inversions to be −0.3 g/cm^3^ (with respect to the reference density of 2.75 g/cm^3^). The upper bound is set to 0.3 g/cm^3^, to account for plutonic rocks of density up to 3.05 g/cm^3^.

### Synthetic tests

We compute forward gravity simulations of a 4 km–by–4 km block located at 6-km depth with a density contrast of 0.5 g/cm^3^ on a finite volume mesh (figs. S3 and S5). We then invert the synthetic data with added gaussian noise (1% of max. gravity value) using nine norm combinations (figs. S4 and S6). Smoother norms (figs. S4 and S6, A to C) result in very small density contrasts over a large area, while more compact norms (fig. S4 and S6, D to I) recover the shape of the blocky source better. The latter are appropriate to represent the storage of melt in the crust, where we expect a clear interface between the density contrasts caused by melt and host rock. The inversion of synthetic VGG data (*g_zz_*) yields more accurate source depth estimates than the inversion of the gravity anomaly (*g_z_*); hence, we use the VGG datasets for all the gravity inversions shown in this study.

## References

[1] R. C. Torres, S. Self, M. M. L. Martinez, C. G. Newhall, R. S. Punongbayan, “Secondary pyroclastic flows from the June 15, 1991, ignimbrite of Mount Pinatubo” in *Fire and Mud: Eruptions and Lahars of Mount Pinatubo, Philippines* (Philippine Institute of Volcanology and Seismology, 1996), pp. 665–678.

[2] W. E. Scott, R. P. Hoblitt, R. C. Torres, S. Self, M. M. L. Martinez, T. Nillos, “Pyroclastic flows of the June 15, 1991, climactic eruption of Mount Pinatubo” in *Fire and Mud: Eruptions and Lahars of Mount Pinatubo, Philippines* (Philippine Institute of Volcanology and Seismology, 1996), pp. 545–570.

[3] J. Vergoz, P. Hupe, C. Listowski, A. Le Pichon, M. A. Garcés, E. Marchetti, P. Labazuy, L. Ceranna, C. Pilger, P. Gaebler, S. P. Näsholm, Q. Brissaud, P. Poli, N. Shapiro, R. De Negri, P. Mialle, IMS observations of infrasound and acoustic-gravity waves produced by the January 2022 volcanic eruption of Hunga, Tonga: A global analysis. Earth Planet. Sci. Lett. 591, 117639 (2022).

[4] R. S. Matoza, D. Fee, J. D. Assink, A. M. Iezzi, D. N. Green, K. Kim, L. Toney, T. Lecocq, S. Krishnamoorthy, J.-M. Lalande, K. Nishida, K. L. Gee, M. M. Haney, H. D. Ortiz, Q. Brissaud, L. Martire, L. Rolland, P. Vergados, A. Nippress, J. Park, S. Shani-Kadmiel, A. Witsil, S. Arrowsmith, C. Caudron, S. Watada, A. B. Perttu, B. Taisne, P. Mialle, A. Le Pichon, J. Vergoz, P. Hupe, P. S. Blom, R. Waxler, S. De Angelis, J. B. Snively, A. T. Ringler, R. E. Anthony, A. D. Jolly, G. Kilgour, G. Averbuch, M. Ripepe, M. Ichihara, A. Arciniega-Ceballos, E. Astafyeva, L. Ceranna, S. Cevuard, I.-Y. Che, R. De Negri, C. W. Ebeling, L. G. Evers, L. E. Franco-Marin, T. B. Gabrielson, K. Hafner, R. G. Harrison, A. Komjathy, G. Lacanna, J. Lyons, K. A. Macpherson, E. Marchetti, K. F. McKee, R. J. Mellors, G. Mendo-Pérez, T. D. Mikesell, E. Munaibari, M. Oyola-Merced, I. Park, C. Pilger, C. Ramos, M. C. Ruiz, R. Sabatini, H. F. Schwaiger, D. Tailpied, C. Talmadge, J. Vidot, J. Webster, D. C. Wilson, Atmospheric waves and global seismoacoustic observations of the January 2022 Hunga eruption, Tonga. Science 377, 95–100 (2022). 35549311 10.1126/science.abo7063

[5] D. A. Yuen, M. A. Scruggs, F. J. Spera, Y. Zheng, H. Hu, S. R. McNutt, G. Thompson, K. Mandli, B. R. Keller, S. S. Wei, Z. Peng, Z. Zhou, F. Mulargia, Y. Tanioka, Under the surface: Pressure-induced planetary-scale waves, volcanic lightning, and gaseous clouds caused by the submarine eruption of Hunga Tonga-Hunga Ha’apai volcano. Earthq. Res. Adv. 2, 100134 (2022).

[6] C. J. Wright, N. P. Hindley, M. J. Alexander, M. Barlow, L. Hoffmann, C. N. Mitchell, F. Prata, M. Bouillon, J. Carstens, C. Clerbaux, S. M. Osprey, N. Powell, C. E. Randall, J. Yue, Surface-to-space atmospheric waves from Hunga Tonga–Hunga Ha’apai eruption. Nature 609, 741–746 (2022). 35772670 10.1038/s41586-022-05012-5 PMC9492537

[7] E. Astafyeva, B. Maletckii, T. D. Mikesell, E. Munaibari, M. Ravanelli, P. Coisson, F. Manta, L. Rolland, The 15 January 2022 Hunga Tonga eruption history as inferred from ionospheric observations. Geophys. Res. Lett. 49, e2022GL098827 (2022).

[8] S. R. Proud, A. T. Prata, S. Schmauß, The January 2022 eruption of Hunga Tonga-Hunga Ha’apai volcano reached the mesosphere. Science 378, 554–557 (2022). 36378963 10.1126/science.abo4076

[9] G. Taha, R. Loughman, P. R. Colarco, T. Zhu, L. W. Thomason, G. Jaross, Tracking the 2022 Hunga Tonga-Hunga Ha’apai aerosol cloud in the upper and middle stratosphere using space-based observations. Geophys. Res. Lett. 49, e2022GL100091 (2022). 10.1029/2022GL100091 PMC9786872 36582258

[10] A. K. Gupta, R. Bennartz, K. E. Fauria, T. Mittal, Eruption chronology of the December 2021 to January 2022 Hunga Tonga-Hunga Ha’apai eruption sequence. Commun. Earth Environ. 3, 314 (2022).

[11] R. Omira, R. S. Ramalho, J. Kim, P. J. González, U. Kadri, J. M. Miranda, F. Carrilho, M. A. Baptista, Global Tonga tsunami explained by a fast-moving atmospheric source. Nature 609, 734–740 (2022). 35697059 10.1038/s41586-022-04926-4 PMC9492550

[12] P. Lynett, M. McCann, Z. Zhou, W. Renteria, J. Borrero, D. Greer, O. Fa’anunu, C. Bosserelle, B. Jaffe, S. La Selle, A. Ritchie, A. Snyder, B. Nasr, J. Bott, N. Graehl, C. Synolakis, B. Ebrahimi, G. E. Cinar, Diverse tsunamigenesis triggered by the Hunga Tonga-Hunga Ha’apai eruption. Nature 609, 728–733 (2022). 35940206 10.1038/s41586-022-05170-6 PMC9472183

[13] S. A. Carn, N. A. Krotkov, B. L. Fisher, C. Li, Out of the blue: Volcanic SO_2_ emissions during the 2021–2022 eruptions of Hunga Tonga—Hunga Ha’apai (Tonga). Front. Earth Sci. 10, 976962 (2022).

[14] L. Millán, M. L. Santee, A. Lambert, N. J. Livesey, F. Werner, M. J. Schwartz, H. C. Pumphrey, G. L. Manney, Y. Wang, H. Su, L. Wu, W. G. Read, L. Froidevaux, The Hunga Tonga-Hunga Ha’apai hydration of the stratosphere. Geophys. Res. Lett. 49, e2022GL099381 (2022). 10.1029/2022GL099381 PMC9285945 35865735

[15] M. A. Clare, I. A. Yeo, S. Watson, R. Wysoczanski, S. Seabrook, K. Mackay, J. E. Hunt, E. Lane, P. J. Talling, E. Pope, S. Cronin, M. Ribó, T. Kula, D. Tappin, S. Henrys, C. de Ronde, M. Urlaub, S. Kutterolf, S. Fonua, S. Panuve, D. Veverka, R. Rapp, V. Kamalov, M. Williams, Fast and destructive density currents created by ocean-entering volcanic eruptions. Science 381, 1085–1092 (2023). 37676954 10.1126/science.adi3038

[16] R. G. Vaughan, P. W. Webley, Satellite observations of a surtseyan eruption: Hunga Ha’apai, Tonga. J. Volcanol. Geotherm. Res. 198, 177–186 (2010).

[17] S. J. Cronin, M. Brenna, I. E. M. Smith, S. J. Barker, M. Tost, M. Ford, S. Tonga’onevai, T. Kula, R. Vaiomounga, “New volcanic island unveils explosive past,” *Eos*, 26 June 2017; http://eos.org/science-updates/new-volcanic-island-unveils-explosive-past.

[18] M. Brenna, S. J. Cronin, I. E. M. Smith, A. Pontesilli, M. Tost, S. Barker, S. Tonga’onevai, T. Kula, R. Vaiomounga, Post-caldera volcanism reveals shallow priming of an intra-ocean arc andesitic caldera: Hunga volcano, Tonga, SW Pacific. Lithos 412–413, 106614 (2022).

[19] K. V. Cashman, G. Giordano, Calderas and magma reservoirs. J. Volcanol. Geotherm. Res. 288, 28–45 (2014).

[20] O. Bachmann, G. Bergantz, The magma reservoirs that feed supereruptions. Elements 4, 17–21 (2008).

[21] D. T. Sandwell, R. D. Müller, W. H. F. Smith, E. Garcia, R. Francis, New global marine gravity model from CryoSat-2 and Jason-1 reveals buried tectonic structure. Science 346, 65–67 (2014). 25278606 10.1126/science.1258213

[22] P. Wessel, Global distribution of seamounts inferred from gridded Geosat/ERS-1 altimetry. J. Geophys. Res. Solid Earth 106, 19431–19441 (2001).

[23] D. T. Sandwell, H. Harper, B. Tozer, W. H. F. Smith, Gravity field recovery from geodetic altimeter missions. Adv. Space Res. 68, 1059–1072 (2021).

[24] R. Cockett, S. Kang, L. J. Heagy, A. Pidlisecky, D. W. Oldenburg, SimPEG: An open source framework for simulation and gradient based parameter estimation in geophysical applications. Comput. Geosci. 85, 142–154 (2015).

[25] C. A. Miller, G. Williams-Jones, D. Fournier, J. Witter, 3D gravity inversion and thermodynamic modelling reveal properties of shallow silicic magma reservoir beneath Laguna del Maule, Chile. Earth Planet. Sci. Lett. 459, 14–27 (2017).

[26] S. F. Trevino, C. A. Miller, B. Tikoff, D. Fournier, B. S. Singer, Multiple, coeval silicic magma storage domains beneath the Laguna Del Maule volcanic field inferred from gravity investigations. J. Geophys. Res. Solid Earth 126, e2020JB020850 (2021).

[27] M. Colombier, B. Scheu, F. B. Wadsworth, S. Cronin, J. Vasseur, K. J. Dobson, K.-U. Hess, M. Tost, T. I. Yilmaz, C. Cimarelli, M. Brenna, B. Ruthensteiner, D. B. Dingwell, Vesiculation and quenching during Surtseyan eruptions at Hunga Tonga-Hunga Ha’apai volcano, Tonga. J. Geophys. Res. Solid Earth 123, 3762–3779 (2018).

[28] I. Ukstins, A. Pontesilli, M. Brenna, J. Wu, S. Cronin, J. Paredes-Mariño, P. Shane, D. Adams, F. Latu’ila, T. Kula, Glass and phenocrysts reveal heterogenous magma sources drove the 15 January 2022 Hunga Volcano eruption, paper presented at IAVCEI 2023 Scientific Assembly, Rotorua, New Zealand, 30 January to 3 February 2023.

[29] F. Caratori Tontini, C. E. J. de Ronde, D. Yoerger, J. Kinsey, M. Tivey, 3-D focused inversion of near-seafloor magnetic data with application to the Brothers volcano hydrothermal system, Southern Pacific Ocean, New Zealand. J. Geophys. Res. Solid Earth 117, B10102 (2012).

[30] F. Caratori Tontini, M. A. Tivey, C. E. J. de Ronde, S. E. Humphris, Heat flow and near-seafloor magnetic anomalies highlight hydrothermal circulation at brothers volcano caldera, Southern Kermadec arc, New Zealand. Geophys. Res. Lett. 46, 8252–8260 (2019).

[31] C. E. J. de Ronde, S. L. Walker, R. G. Ditchburn, F. C. Tontini, M. D. Hannington, S. G. Merle, C. Timm, M. R. Handler, R. J. Wysoczanski, V. M. Dekov, G. D. Kamenov, E. T. Baker, R. W. Embley, J. E. Lupton, P. Stoffers, The anatomy of a buried submarine hydrothermal system, Clark volcano, Kermadec arc, New Zealand. Econ. Geol. 109, 2261–2292 (2014).

[32] S. Scott, T. Driesner, P. Weis, The thermal structure and temporal evolution of high-enthalpy geothermal systems. Geothermics 62, 33–47 (2016).

[33] C. Huber, M. Townsend, W. Degruyter, O. Bachmann, Optimal depth of subvolcanic magma chamber growth controlled by volatiles and crust rheology. Nat. Geosci. 12, 762–768 (2019).

[34] D. J. Rasmussen, T. A. Plank, D. C. Roman, M. M. Zimmer, Magmatic water content controls the pre-eruptive depth of arc magmas. Science 375, 1169–1172 (2022). 35271312 10.1126/science.abm5174

[35] M. E. Pritchard, P. M. Gregg, Geophysical evidence for silicic crustal melt in the continents: Where, what kind, and how much? Elements 12, 121–127 (2016).

[36] C. Magee, C. T. E. Stevenson, S. K. Ebmeier, D. Keir, J. O. S. Hammond, J. H. Gottsmann, K. A. Whaler, N. Schofield, C. A.-L. Jackson, M. S. Petronis, B. O’Driscoll, J. Morgan, A. Cruden, S. A. Vollgger, G. Dering, S. Micklethwaite, M. D. Jackson, Magma plumbing systems: A geophysical perspective. J. Petrol. 59, 1217–1251 (2018).

[37] G. Weber, L. Caricchi, J. L. Arce, A. K. Schmitt, Determining the current size and state of subvolcanic magma reservoirs. Nat. Commun. 11, 5477 (2020). 33154361 10.1038/s41467-020-19084-2 PMC7644707

[38] J. Korenaga, P. B. Kelemen, Melt migration through the oceanic lower crust: A constraint from melt percolation modeling with finite solid diffusion. Earth Planet. Sci. Lett. 156, 1–11 (1998).

[39] K. V. Cashman, R. S. J. Sparks, J. D. Blundy, Vertically extensive and unstable magmatic systems: A unified view of igneous processes. Science 355, eaag3055 (2017). 28336610 10.1126/science.aag3055

[40] B. D. Marsh, On the crystallinity, probability of occurrence, and rheology of lava and magma. Contr. to Mineral. Petrol. 78, 85–98 (1981).

[41] M. M. Yale, D. T. Sandwell, A. T. Herring, What are the limitations of satellite altimetry? Lead. Edge 17, 73–76 (1998).

[42] M. P. Poland, D. Carbone, M. R. Patrick, Onset and evolution of Kīlauea’s 2018 flank eruption and summit collapse from continuous gravity. Earth Planet. Sci. Lett. 567, 117003 (2021).

[43] S. M. Carbotte, A. Arnulf, M. Spiegelman, M. Lee, A. Harding, G. Kent, J. P. Canales, M. Nedimović, Stacked sills forming a deep melt-mush feeder conduit beneath Axial Seamount. Geology 48, 693–697 (2020).

[44] A. F. Arnulf, A. J. Harding, G. M. Kent, W. S. D. Wilcock, Structure, seismicity, and accretionary processes at the hot spot-influenced Axial Seamount on the Juan de Fuca Ridge. J. Geophys. Res. Solid Earth 123, 4618–4646 (2018).

[45] O. Foix, C. Aiken, J.-M. Saurel, N. Feuillet, S. J. Jorry, E. Rinnert, I. Thinon, Offshore Mayotte volcanic plumbing revealed by local passive tomography. J. Volcanol. Geotherm. Res. 420, 107395 (2021).

[46] B. G. McVey, E. E. E. Hooft, B. A. Heath, D. R. Toomey, M. Paulatto, J. V. Morgan, P. Nomikou, C. B. Papazachos, Magma accumulation beneath Santorini volcano, Greece, from P-wave tomography. Geology 48, 231–235 (2020).

[47] K. Chrapkiewicz, M. Paulatto, B. A. Heath, E. E. E. Hooft, P. Nomikou, C. B. Papazachos, F. Schmid, D. R. Toomey, M. R. Warner, J. V. Morgan, Magma chamber detected beneath an arc volcano with full-waveform inversion of active-source seismic data. Geochem. Geophys. Geosyst. 23, e2022GC010475 (2022).

[48] B. M. Kilbride, M. Edmonds, J. Biggs, Observing eruptions of gas-rich compressible magmas from space. Nat. Commun. 7, 13744 (2016). 28000791 10.1038/ncomms13744 PMC5187499

[49] M. Ribó, S. Cronin, S. Stern, S.-H. Park, J. Garvin, T. Kula, F. H. Latu’ila, P. Tukafu, N. Heni, Detailed morphology of the Hunga Tonga-Hunga Ha’apai caldera: Evolution of the submarine volcano after the explosive eruption, paper presented at IAVCEI 2023 Scientific Assembly, Rotorua, New Zealand, 30 January to 3 February 2023.

[50] A. Geyer, A. Folch, J. Martí, Relationship between caldera collapse and magma chamber withdrawal: An experimental approach. J. Volcanol. Geotherm. Res. 157, 375–386 (2006).

[51] K. R. Anderson, I. A. Johanson, M. R. Patrick, M. Gu, P. Segall, M. P. Poland, E. K. Montgomery-Brown, A. Miklius, Magma reservoir failure and the onset of caldera collapse at Kīlauea Volcano in 2018. Science 366, eaaz1822 (2019). 31806783 10.1126/science.aaz1822

[52] M. T. Gudmundsson, K. Jónsdóttir, A. Hooper, E. P. Holohan, S. A. Halldórsson, B. G. Ófeigsson, S. Cesca, K. S. Vogfjörd, F. Sigmundsson, T. Högnadóttir, P. Einarsson, O. Sigmarsson, A. H. Jarosch, K. Jónasson, E. Magnússon, S. Hreinsdóttir, M. Bagnardi, M. M. Parks, V. Hjörleifsdóttir, F. Pálsson, T. R. Walter, M. P. J. Schöpfer, S. Heimann, H. I. Reynolds, S. Dumont, E. Bali, G. H. Gudfinnsson, T. Dahm, M. J. Roberts, M. Hensch, J. M. C. Belart, K. Spaans, S. Jakobsson, G. B. Gudmundsson, H. M. Fridriksdóttir, V. Drouin, T. Dürig, G. Aðalgeirsdóttir, M. S. Riishuus, G. B. M. Pedersen, T. van Boeckel, B. Oddsson, M. A. Pfeffer, S. Barsotti, B. Bergsson, A. Donovan, M. R. Burton, A. Aiuppa, Gradual caldera collapse at Bárdarbunga volcano, Iceland, regulated by lateral magma outflow. Science 353, (2016). 10.1126/science.aaf8988 27418515

[53] B. Riel, P. Milillo, M. Simons, P. Lundgren, H. Kanamori, S. Samsonov, The collapse of Bárðarbunga caldera, Iceland. Geophys. J. Int. 202, 446–453 (2015).

[54] A. H. Lerner, D. O’Hara, L. Karlstrom, S. K. Ebmeier, K. R. Anderson, S. Hurwitz, The prevalence and significance of offset magma reservoirs at arc volcanoes. Geophys. Res. Lett. 47, e2020GL087856 (2020).

[55] D. Cordell, M. J. Unsworth, D. Díaz, Imaging the Laguna del Maule Volcanic Field, central Chile using magnetotellurics: Evidence for crustal melt regions laterally-offset from surface vents and lava flows. Earth Planet. Sci. Lett. 488, 168–180 (2018).

[56] S. K. Ebmeier, B. J. Andrews, M. C. Araya, D. W. D. Arnold, J. Biggs, C. Cooper, E. Cottrell, M. Furtney, J. Hickey, J. Jay, R. Lloyd, A. L. Parker, M. E. Pritchard, E. Robertson, E. Venzke, J. L. Williamson, Synthesis of global satellite observations of magmatic and volcanic deformation: Implications for volcano monitoring & the lateral extent of magmatic domains. J. Appl. Volcanol. 7, 2 (2018).

[57] T. Mittal, J. S. Jordan, L. Retailleau, F. Beauducel, A. Peltier, Mayotte 2018 eruption likely sourced from a magmatic mush. Earth Planet. Sci. Lett. 590, 117566 (2022).

[58] J. Wu, I. Ukstins, S. Cronin, D. Adams, A. Klein, J. Vongsvivut, J. Paredes-Mariño, Magmatic volatiles in the 15th January 2022 Hunga volcano, Tonga, paper presented at IAVCEI 2023 Scientific Assembly, Rotorua, New Zealand, 30 January to 3 February 2023.

[59] F. Ramos, P. A. Shane, I. Ukstins, D. Adams, W. Jui, J. Paredes Marino, S. Cronin, M. Brenna, F. H. Latu’ila, T. Kula, The Isotopic Nature of the Magmas and Crystals Involved in the 2021-22 Hunga Volcano Eruption, paper presented at IAVCEI 2023 Scientific Assembly, Rotorua, New Zealand, 30 January to 3 February 2023.

[60] J. A. Crisp, Rates of magma emplacement and volcanic output. J. Volcanol. Geotherm. Res. 20, 177–211 (1984).

[61] E. S. Garcia, D. T. Sandwell, W. H. F. Smith, Retracking CryoSat-2, Envisat and Jason-1 radar altimetry waveforms for improved gravity field recovery. Geophys. J. Int. 196, 1402–1422 (2014).

[62] D. Sandwell, E. Garcia, K. Soofi, P. Wessel, M. Chandler, W. H. F. Smith, Toward 1-mGal accuracy in global marine gravity from CryoSat-2, Envisat, and Jason-1. Lead. Edge 32, 892–899 (2013).

[63] D. T. Sandwell, W. H. F. Smith, Global marine gravity from retracked Geosat and ERS-1 altimetry: Ridge segmentation versus spreading rate. J. Geophys. Res. Solid Earth 114, 10.1029/2008JB006008, (2009).

[64] R. L. Parker, The rapid calculation of potential anomalies. Geophys. J. Int. 31, 447–455 (1973).

[65] P. Wessel, W. H. F. Smith, R. Scharroo, J. Luis, F. Wobbe, Generic Mapping Tools: Improved version released. Eos. Transactions American Geophysical Union 94, 409–410 (2013).

[66] D. Fournier, D. W. Oldenburg, Inversion using spatially variable mixed *𝓁_p_* norms. Geophys. J. Int. 218, 268–282 (2019).

[67] C. DeMets, R. G. Gordon, D. F. Argus, Geologically current plate motions. Geophys. J. Int. 181, 1–80 (2010).

[68] J. B. Garvin, D. A. Slayback, V. Ferrini, J. Frawley, C. Giguere, G. R. Asrar, K. Andersen, Monitoring and modeling the rapid evolution of Earth’s newest volcanic island: *Hunga Tonga Hunga Ha’apai* (Tonga) using high spatial resolution satellite observations. Geophys. Res. Lett. 45, 3445–3452 (2018). 30034048 10.1002/2017GL076621 PMC6049963

