# Supplementary material for: The magmatic system under Hunga volcano before and after the 15 January 2022 eruption
Source: Sci Adv. 2023 Dec 15;9(50):eadh3156. doi: 10.1126/sciadv.adh3156 (PMC10848737; doi:10.1126/sciadv.adh3156)
Supplement: Supplementary file 1 — Figs. S1 to S21 Legends for data S1 and S2 [file sciadv.adh3156_sm.pdf]

Science Advances
AAAS

Supplementary Materials for

The magmatic system under Hunga volcano before and after the 15 January 2022 eruption

Hélène Le Mével *et al.*

Corresponding author: Hélène Le Mével, hlemevel@carnegiescience.edu

Sci. Adv. **9**, eadh3156 (2023)
DOI: 10.1126/sciadv.adh3156

The PDF file includes:

Figs. S1 to S21
Legends for data S1 and S2

Other Supplementary Material for this manuscript includes the following:

Data S1 and S2

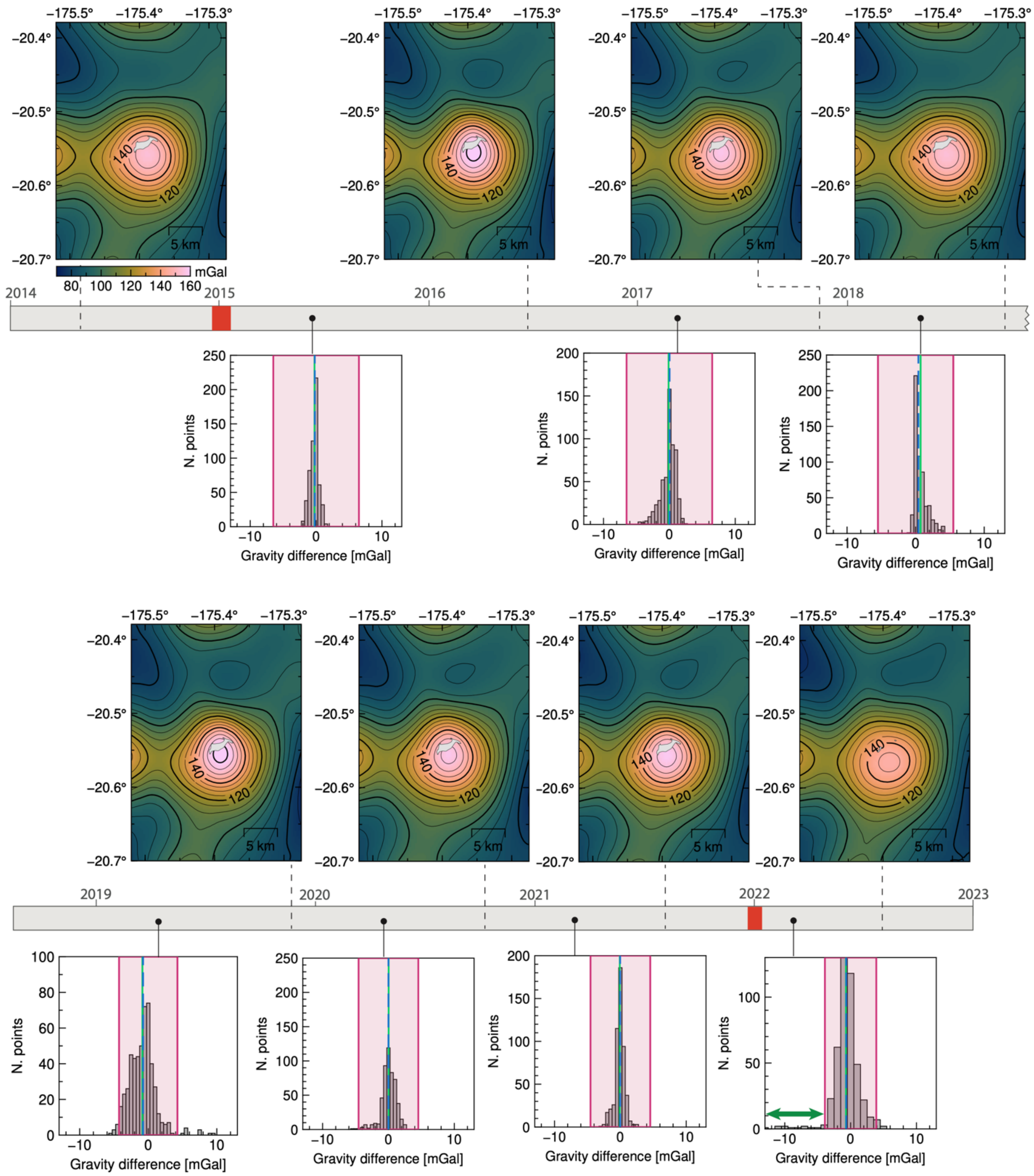

Fig. S1. Timeline of marine gravity anomaly. Annual updates of the marine gravity grid (colorbar) show that no significant change is observed until after the 2022 eruption. Histograms of the yearly difference and uncertainty (pink) estimated for the difference in gravity anomaly. Eruptive episodes shown as red bar. Green arrow on last histogram shows the significant gravity decrease observed between August 2021 and August 2022.

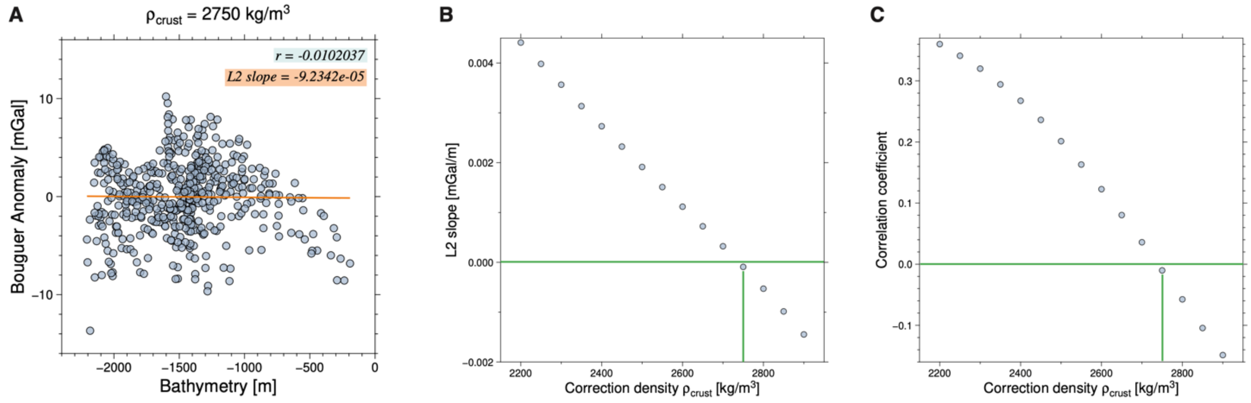

Fig. S2. Choice of crustal correction density for the Bouguer anomaly calculation. (A) Relationship between residual Bouguer anomaly and bathymetry for the optimal correction density of 2750 kg/m^3 and best fit least-square regression (orange line). **(B)** Least-square slope estimates (*L2 slope*), and **(C)** Pearson correlation coefficient (*r*) for correction density varying from 2200 to 2900 kg/m^3. The correlation is minimized (closest to 0, shown as green line) for a correction density of 2750 kg/m^3.

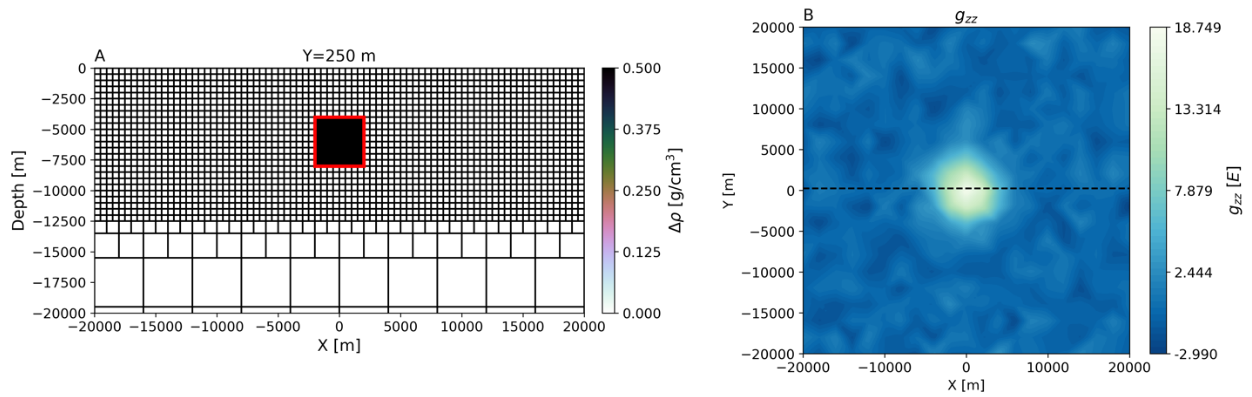

Fig. S3. Synthetic block model for the VGG inversion. (A) Model mesh for a 4 km by 4 km prism buried at 6 km depth with $\Delta\rho = 0.5$ g/cm^3. **(B)** Synthetic vertical gravity gradient (VGG) data due to the density anomaly in (A) with the addition of gaussian noise (1% of the maximum anomaly amplitude).

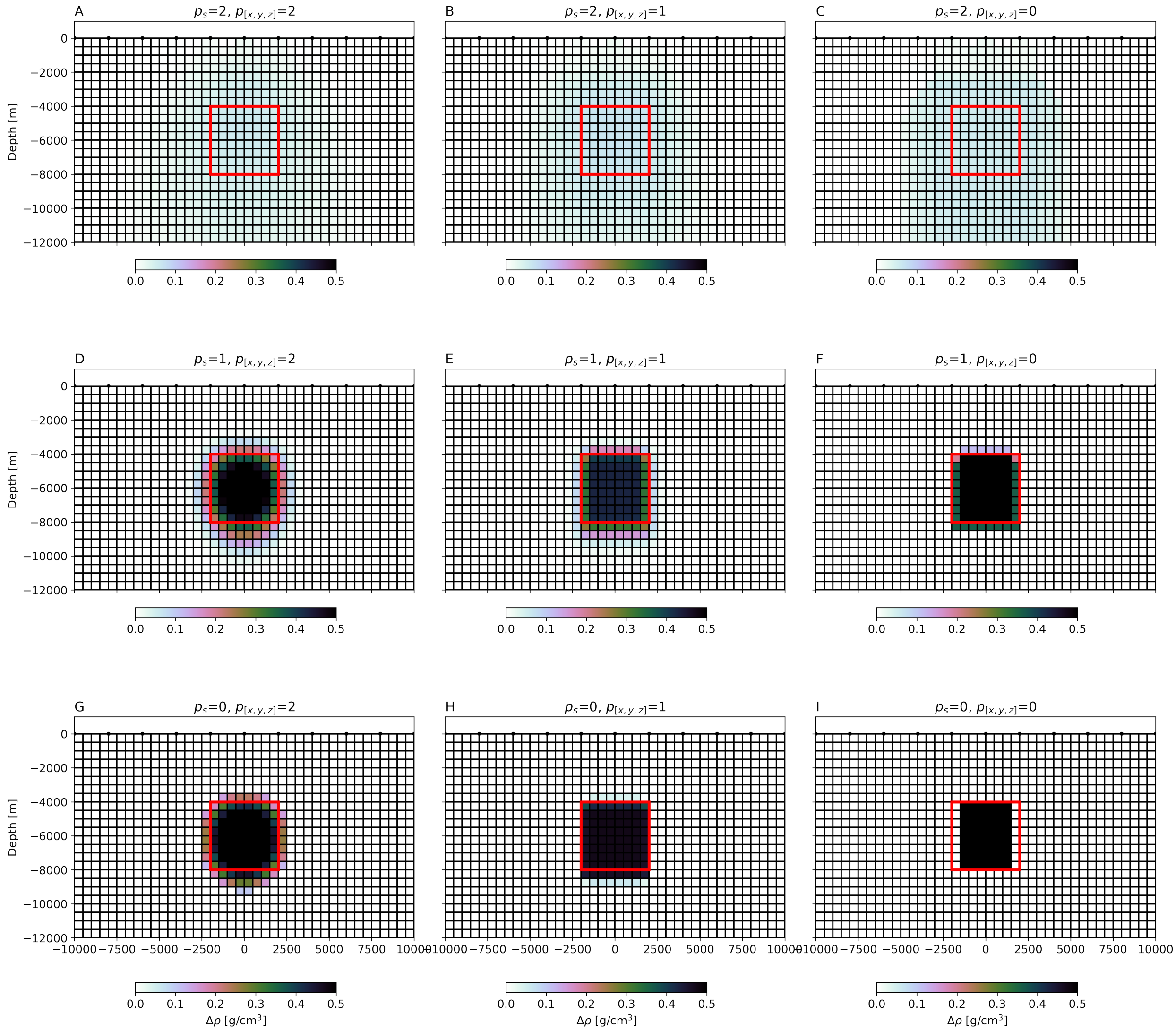

Fig. S4. Density contrast recovery for a VGG synthetic model of a 4 km by 4 km prism at 6 km depth (Fig. S3) with Δρ = 0.5 g/cm³, for different combinations of inversion norms (smoothest, A, to most compact, I) using SimPEG. The inversions with the smoothest norms (A to C) attribute a very small density contrast to a large area and fail to recover the prism depth, shape, and density contrast, therefore we do not consider them for our data inversions. Preferred norms used in modeling Hunga volcano data are D, E, and G (Fig. S7 to S13).

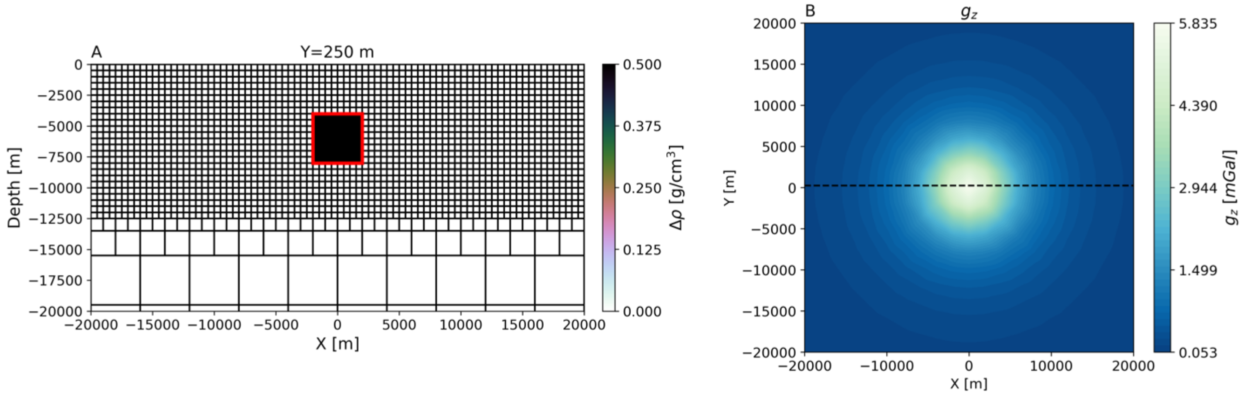

Fig. S5. Synthetic block model for the gravity (g_z) anomaly inversion. (A) Model mesh for a 4 km by 4 km prism at 6 km depth with $\Delta\rho = 0.5$ g/cm^3. **(B)** Synthetic gravity data due to the density anomaly in (A) with the addition of gaussian noise (1% of the maximum anomaly amplitude).

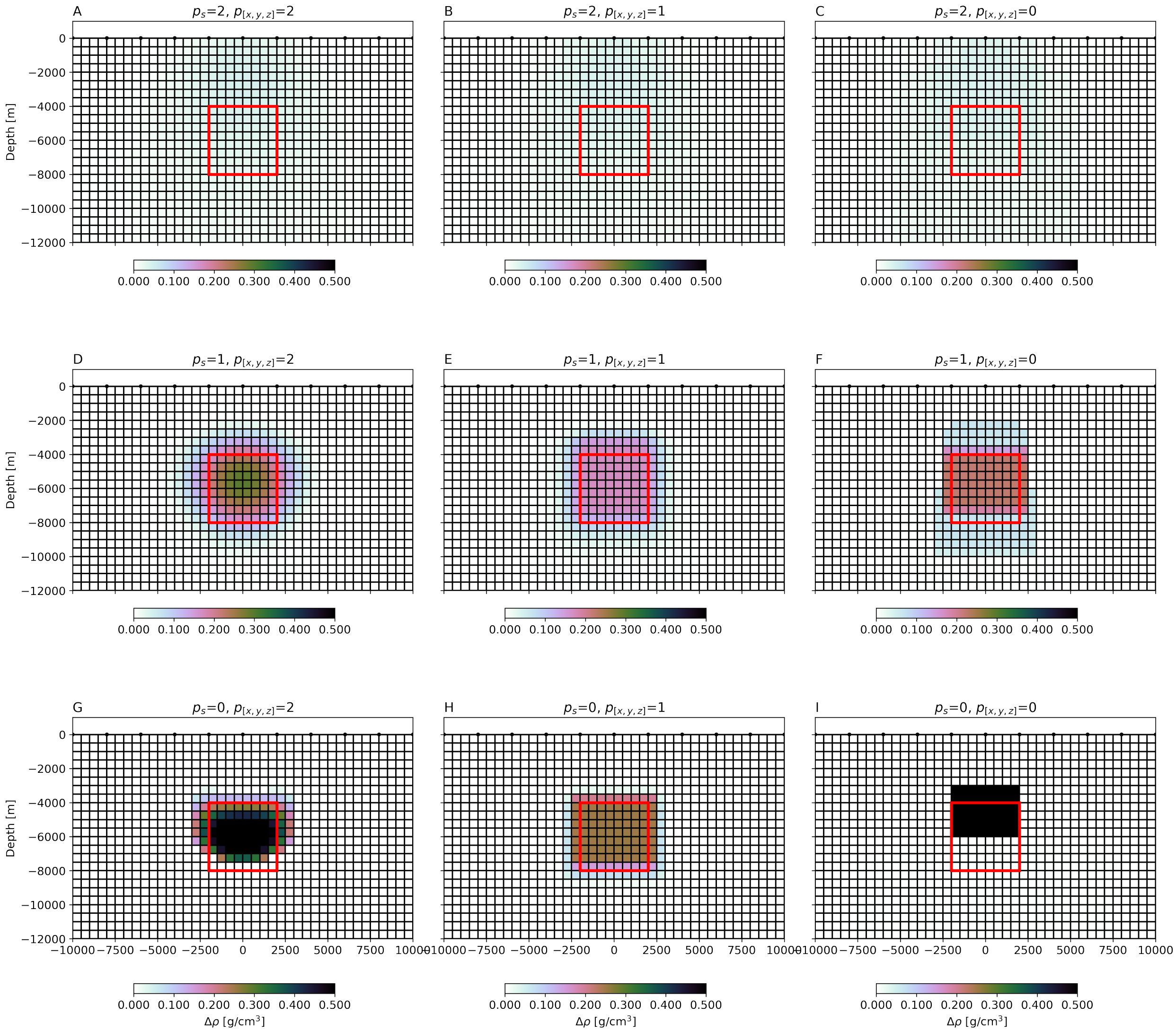

Fig. S6. Density contrast recovery for the Bouguer gravity anomaly synthetic model of a 4 km by 4 km prism at 6 km depth (Fig. S4) with $\Delta\rho = 0.5$ g/cm^3, for different combinations of inversion norms (smoothest, A, to most compact, I) using SimPEG. Source depth, shape, and density contrast recovery are more accurate for VGG inversions (Fig. S4) therefore we use VGG data to model Hunga volcano gravity anomalies.

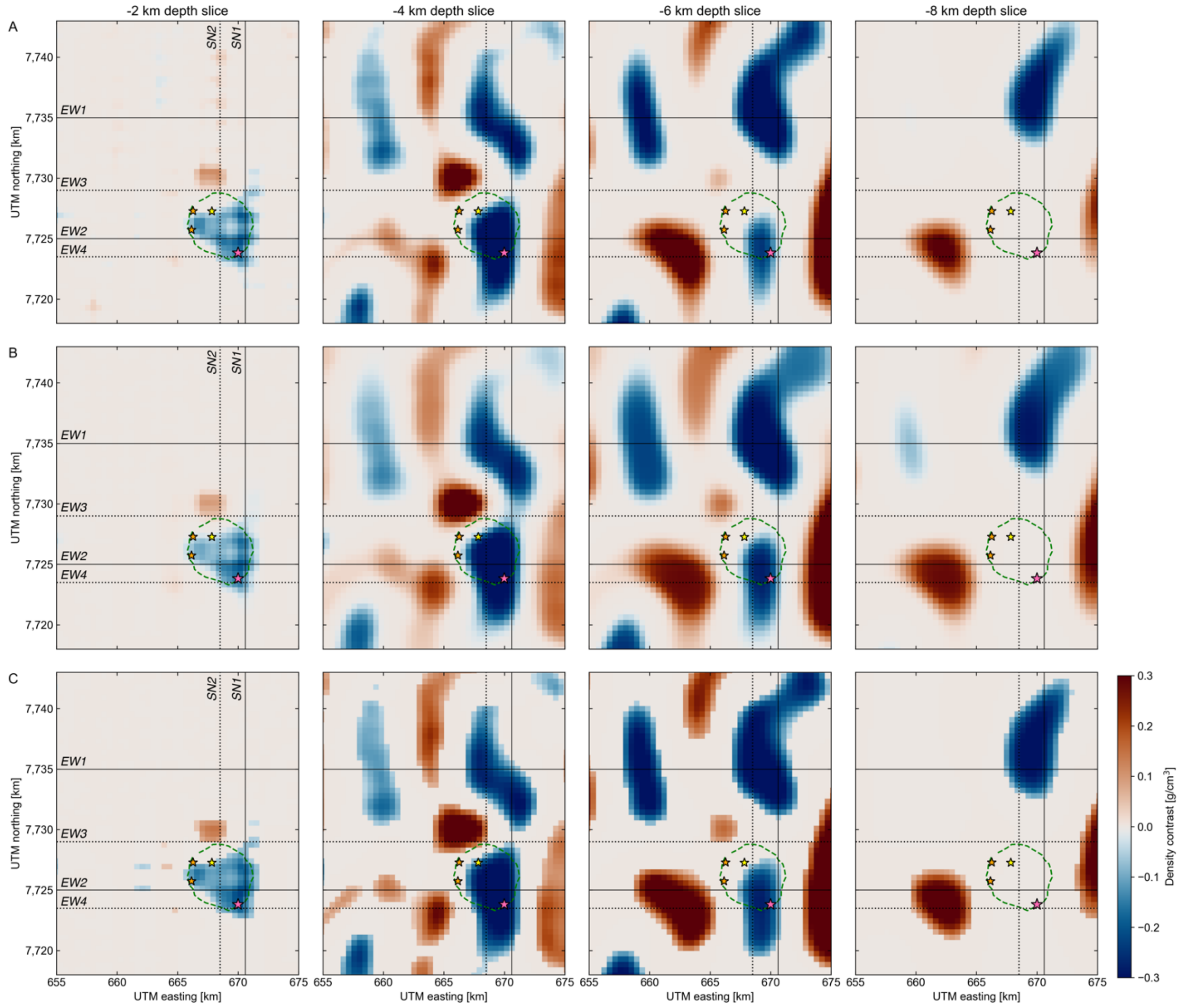

Fig. S7. Depth slices through the 3D density model of the pre-eruptive magmatic system. Inversion results with three different norm combinations: [1,2,2,2] **(A)**, [1,1,1,1] **(B)**, and [0,2,2,2] **(C)**. Color scale shows the density contrast with respect to a reference density of 2.75 g/cm^3. Caldera outline (dashed green line) and historical vents (stars). Thin black lines and dotted lines are model cross sections displayed on Fig. S8 and Figures 4 and 5 in the main manuscript.

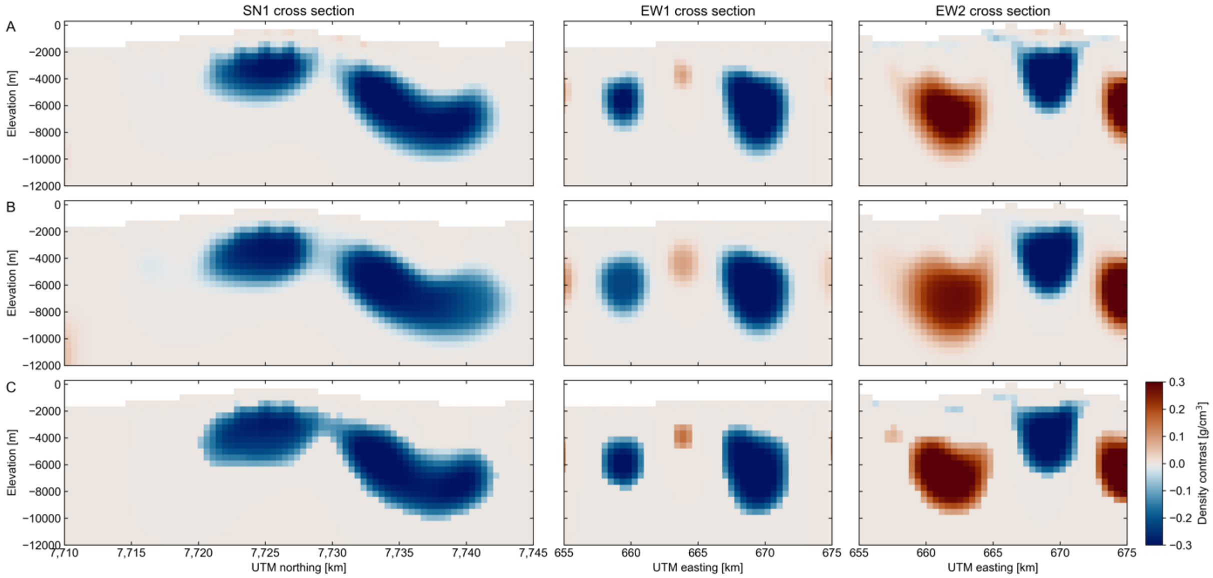

Fig. S8. Cross sections through the 3D density model of the pre-eruptive magmatic system. **(A)**, **(B)** and **(C)** are inversion results with three different norm combinations: [1,2,2,2], [1,1,1,1], and [0,2,2,2] respectively. Color scale shows the density contrast with respect to a reference density of 2.75 g/cm^3. Location of cross sections are shown on Fig. S7.

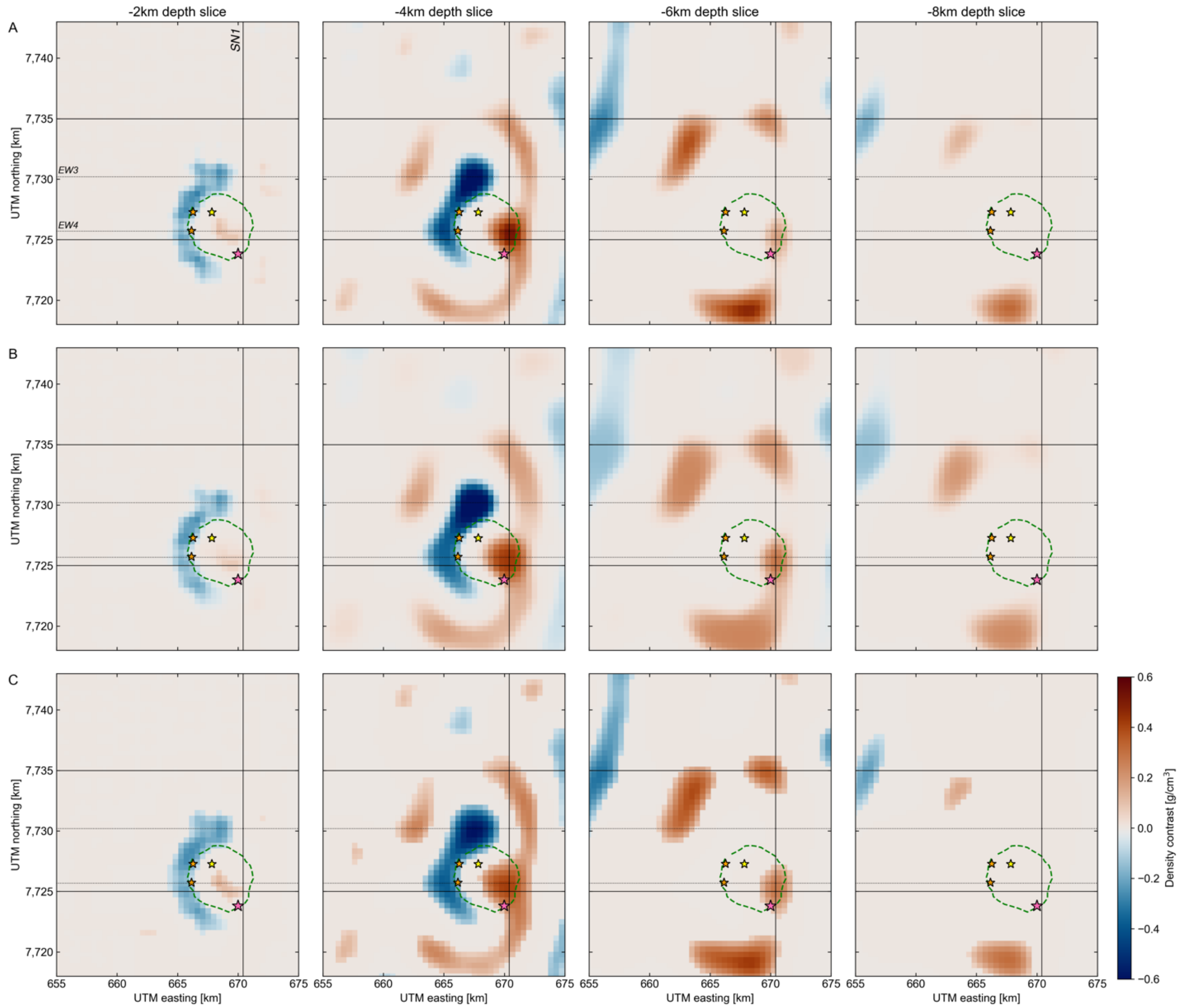

Fig. S9. Depth slices through the 3D density model of the co-eruptive magmatic system. Inversion results with three different norm combinations: [1,2,2,2] **(A)**, [1,1,1,1] **(B)**, and [0,2,2,2] **(C)**. Color scale shows the density contrast with respect to a reference density of 2.75 g/cm^3. Caldera outline (dashed green line) and historical vents (stars). Thin black lines and dotted lines are model cross sections displayed on Fig. S10.

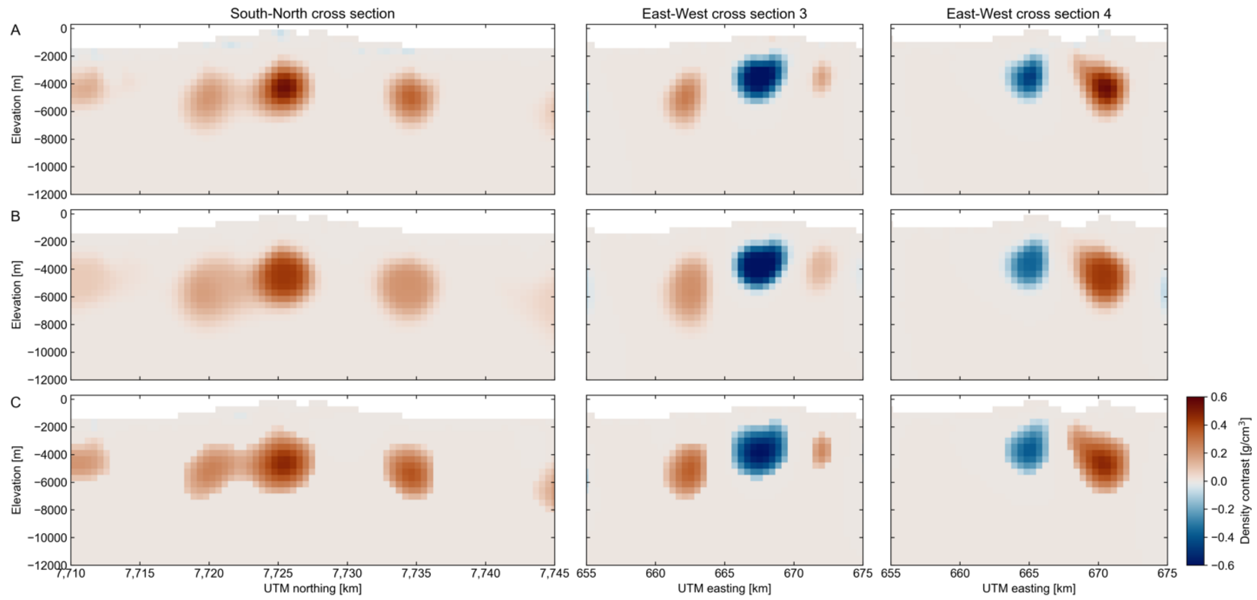

Fig. S10. Cross sections through the 3D density model of the co-eruptive magmatic system. **(A)**, **(B)** and **(C)** are inversion results with three different norm combinations: [1,2,2,2], [1,1,1,1], and [0,2,2,2] respectively. Color scale shows the density contrast with respect to a reference density of 2.75 g/cm^3. Location of cross sections are shown on Fig. S9.

Before		After		Processes	Change
--	-	-	0	Melt movement creating new void replaced by rocks (e.g. after collapse), decrease in melt fraction due to solidification	Mass/density increase
--	-	+	++	Syn-eruptive crystallization due to rapid decompression of magma reservoir, pressure-quenching of mush	
0	+	+	++	Emplacement of denser material into the crust, reorganization of crustal rocks	
+	++	0	+	Increase in melt fraction due to addition of fluids or magmatic melt	Mass/density decrease
-	0	--	-	Addition of fluids or magmatic melt, increasing pore space e.g. due to faulting	
+	++	--	-	Increase in pore space, development of fractures and/or addition of fluids	

Figure S11. Interpretation of gravity changes. Possible physical processes explaining the mass/density increase or decrease depending on the sign of the observed gravity anomaly before and after the eruption. Blue -/-- indicates weak/strong negative anomaly, Red +/++ indicates weak/strong positive anomaly. 0 indicates no gravity anomaly.

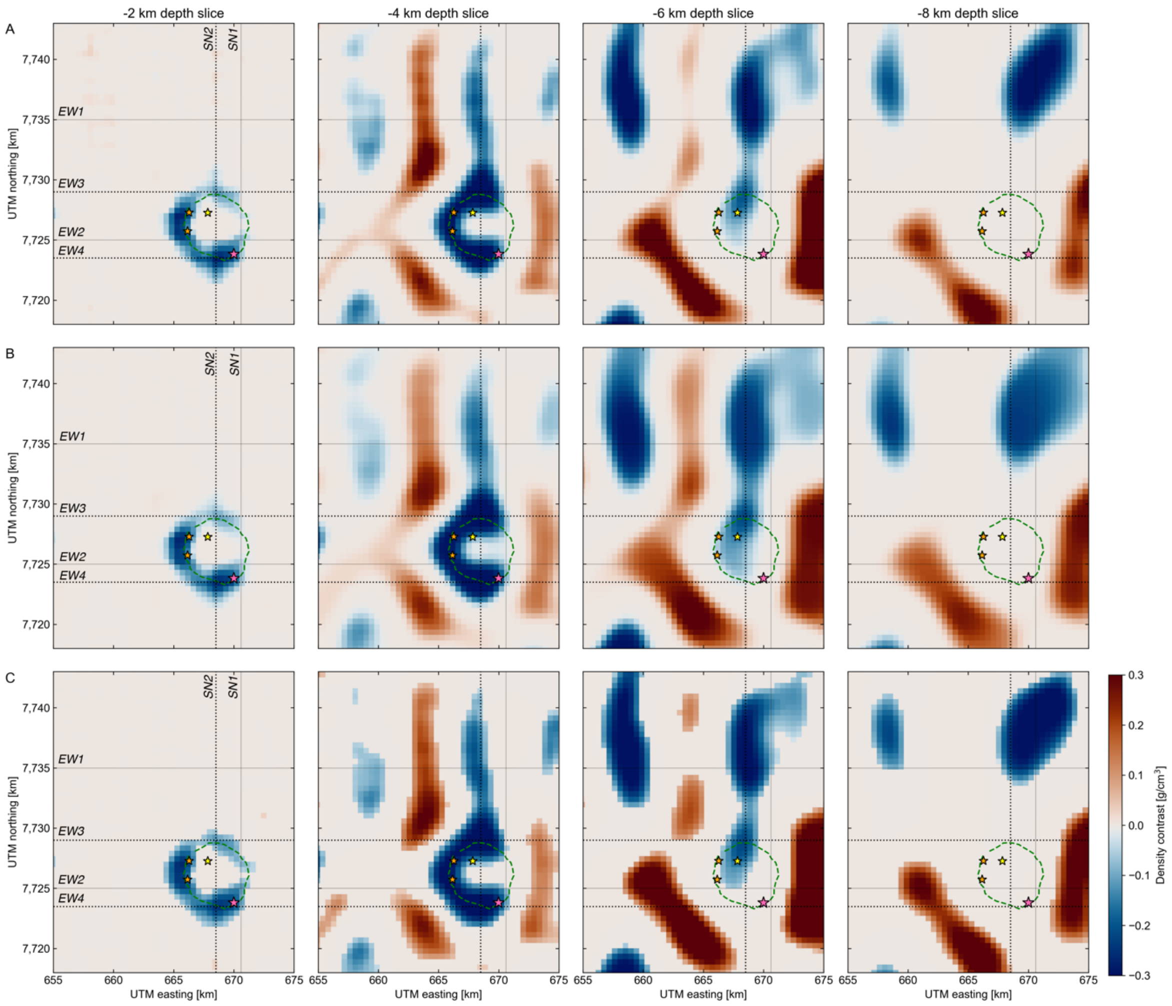

Fig. S12. Depth slices through the 3D density model of the post-eruptive magmatic system. Inversion results with three different norm combinations: [1,2,2,2] **(A)**, [1,1,1,1] **(B)**, and [0,2,2,2] **(C)**. Color scale shows the density contrast with respect to a reference density of 2.75 g/cm^3. Caldera outline (dashed green line) and historical vents (stars). Thin black lines are the same as in Fig. S6 (shown for reference only), and dotted lines are model cross sections displayed on Fig. S13 and Figures 4 and 5 in the main manuscript.

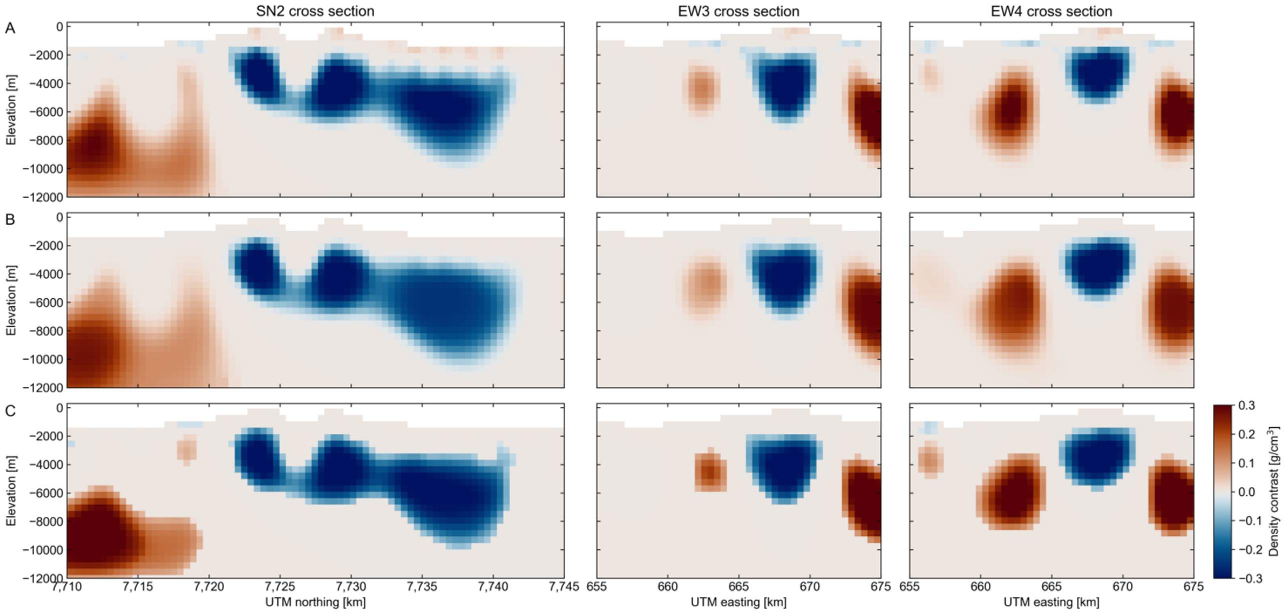

Fig. S13. Cross sections through the 3D density model of the post-eruptive magmatic system. (A), **(B)** and **(C)** are inversion results with three different norm combinations: [1,2,2,2], [1,1,1,1], and [0,2,2,2], respectively. Color scale shows the density contrast with respect to a reference density of 2.75 g/cm^3. Location of cross sections are shown on Fig. S12 (dotted black lines).

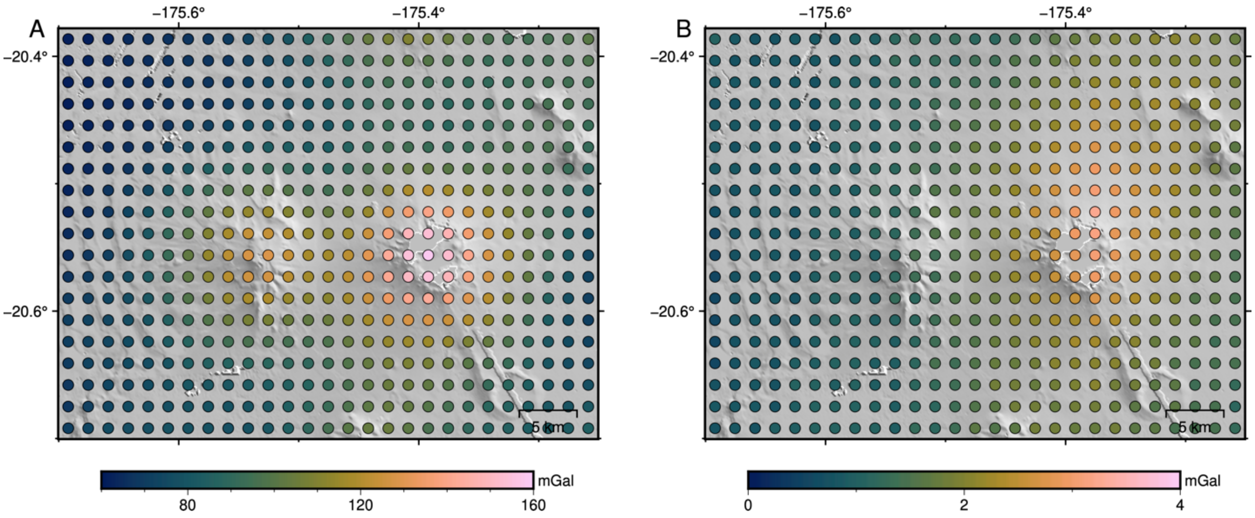

Fig. S14. Pre-eruptive gravity data and uncertainty. (A) 1-arcminute marine gravity grid from satellite altimetry (V31.1) (*21, 23*) (colored circles) superimposed on shaded relief of the pre-eruptive multibeam bathymetry. **(B)** Uncertainty on the marine gravity anomaly varies from 0.6 to 3.2 mGal with a mean of 1.5 mGal over the study area.

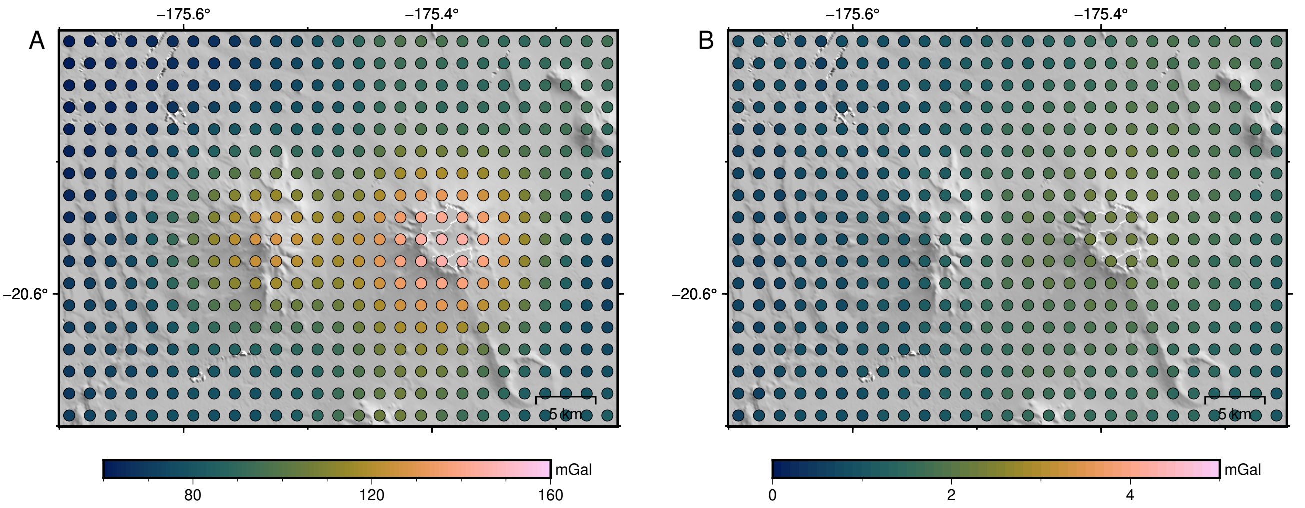

Fig. S15. Post-eruptive gravity data and uncertainty. (A) 1-arcminute marine gravity grid from satellite altimetry (V32.1) (*21, 23*) for the post-eruptive time interval (colored circles) superimposed on shaded relief of the pre-eruptive multibeam bathymetry. **(B)** Uncertainty on the marine gravity anomaly varies from 0.6 to 2.2 mGal with a mean of 1.3 mGal over the study area.

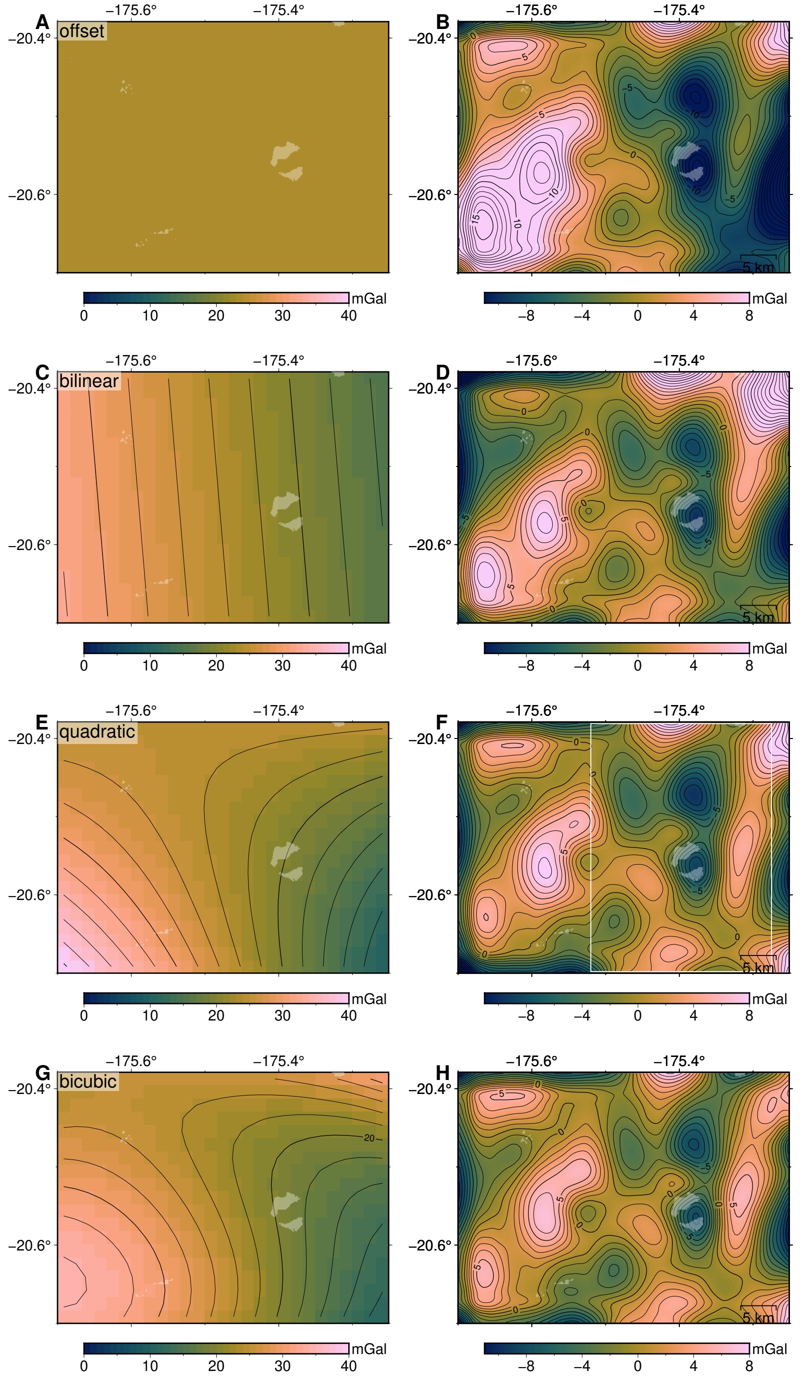

Fig. S16. Comparison of regional trends estimated from the Bouguer anomaly (reference density 2750 kg/m^3) using a polynomial of degree: **(A)** 0 (constant offset), **(C)** 1 (bilinear, 3 terms), **(E)** 2 (quadratic, 6 terms), and **(G)** 3 (bicubic, 10 terms). **(B)**, **(D)**, **(F)**, and **(H)** show the residual detrended Bouguer anomaly for each trend type. The quadratic polynomial **(F)** with 6 model parameters ($\boldsymbol{m}$) follows the equation: $\boldsymbol{m_1 + m_2x + m_3y + m_4xy + m_5x^2 + m_6x^2}$ and is chosen to approximate the regional gravity field. White frame indicates the region shown on Figure 3.

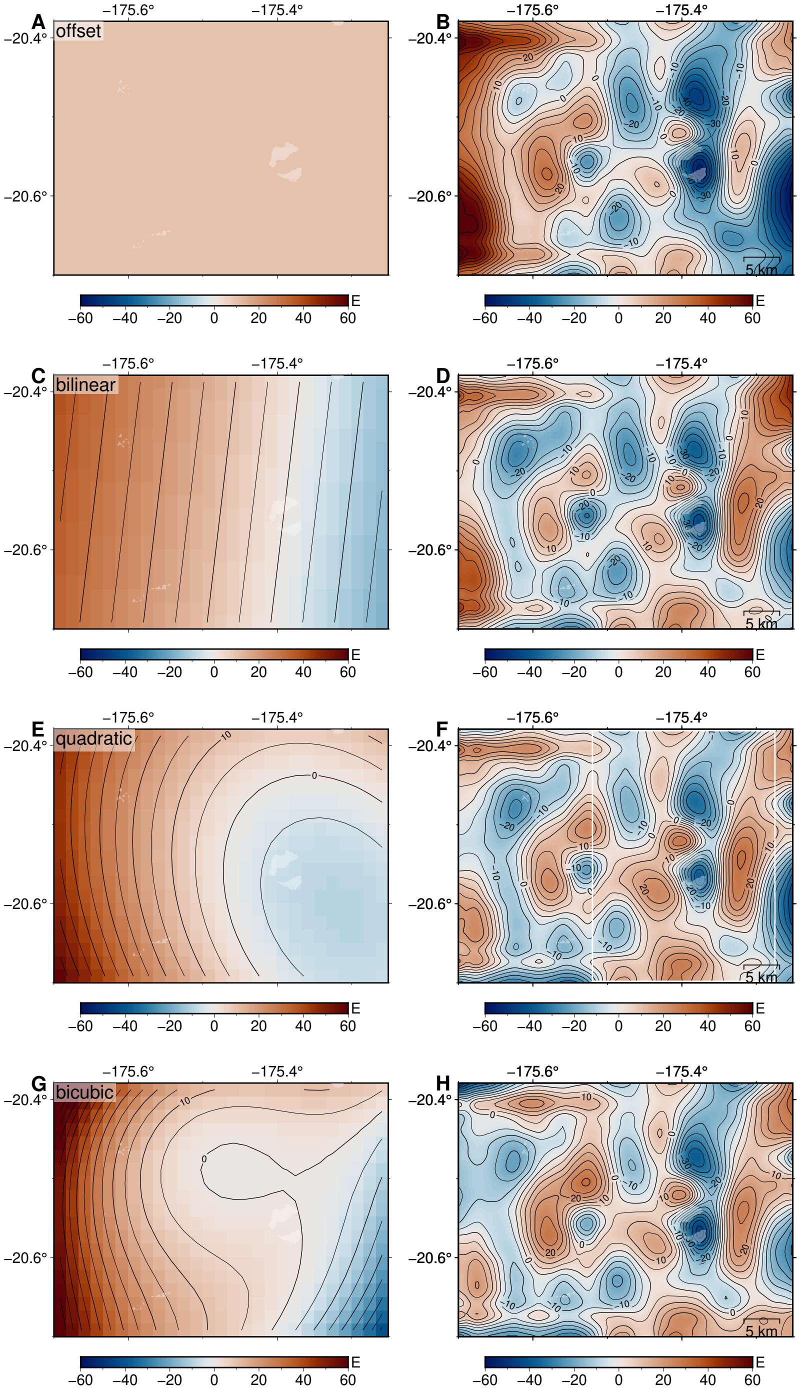

Fig. S17. Comparison of regional trends estimated from the VGG-derived Bouguer anomaly (reference density 2750 kg/m^3) using a polynomial of degree: **(A)** 0 (constant offset), **(C)** 1 (bilinear, 3 terms), **(E)** 2 (quadratic, 6 terms), and **(G)** 3 (bicubic, 10 terms). **(B)**, **(D)**, **(F)**, and **(H)** show the residual detrended Bouguer anomaly for each trend type. The quadratic polynomial **(F)** with 6 model parameters ($\boldsymbol{m}$) follows the equation: $\boldsymbol{m_1 + m_2x + m_3y + m_4xy + m_5x^2 + m_6x^2}$ and is chosen to approximate the regional gravity field. White frame indicates the region shown on Figure 3.

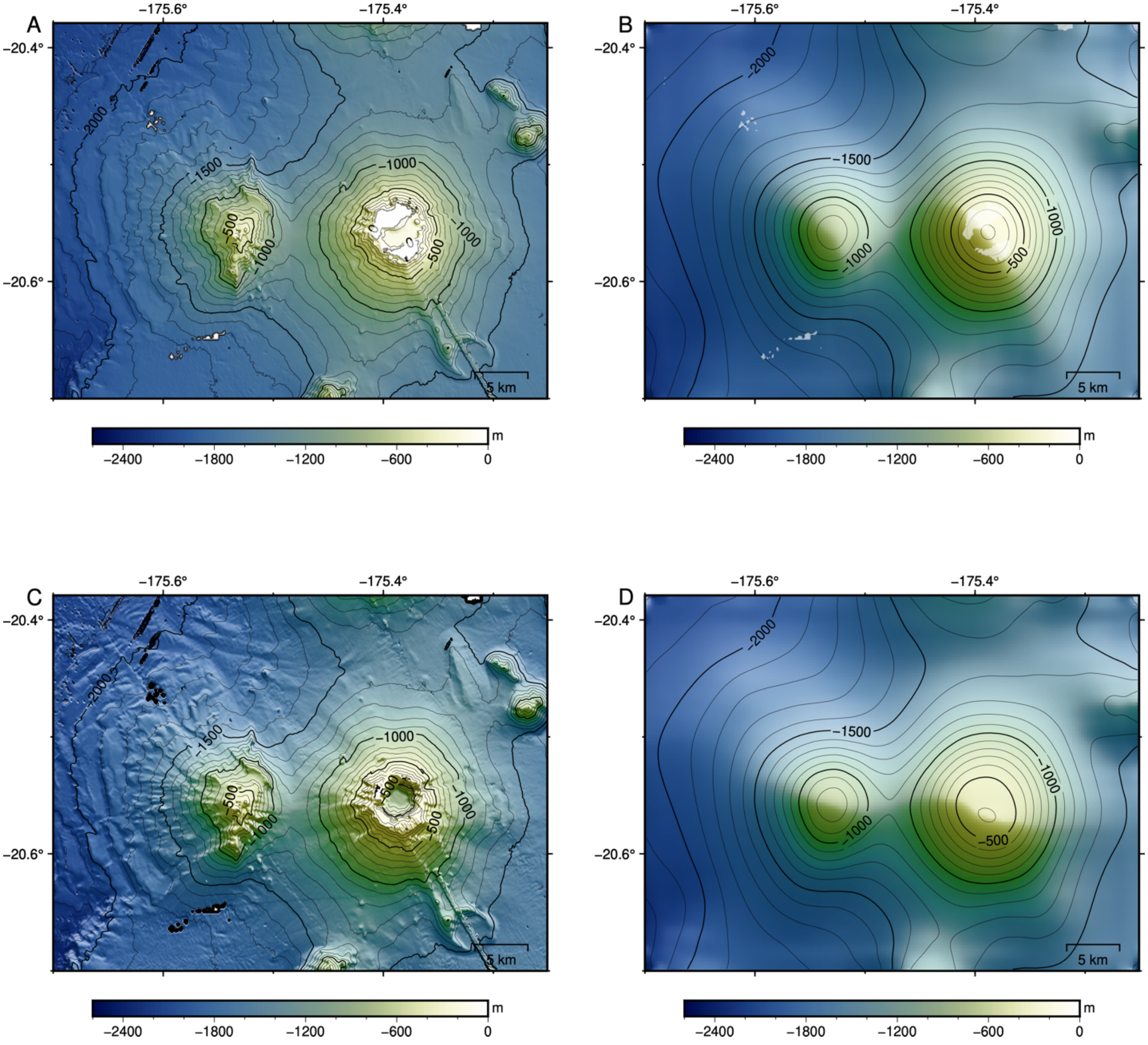

Fig. S18. Downsampling of bathymetry grids. (A) Pre-eruptive bathymetry grid with original 3-arcsecond resolution, **(B)** downsampled to 1-arcminute resolution using a Gaussian filter (standard deviation of 2 km), **(C)** post-eruptive bathymetry grid, and **(D)** downsampled to 1-arcminute resolution used for the calculation of the gravity effect of the bathymetry interface to derive the Bouguer anomaly.

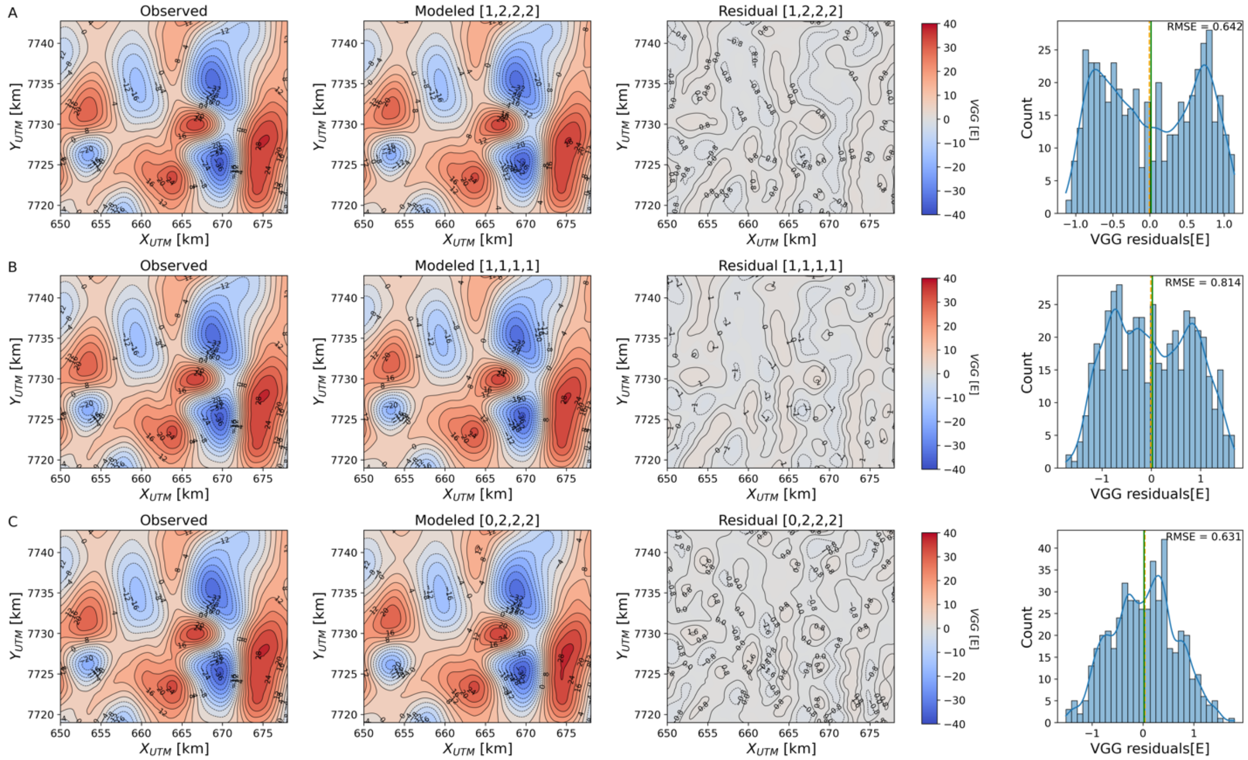

Fig. S19. Model misfit for the pre-eruptive VGG Bouguer anomaly. Observed VGG anomaly, modeled, and residuals for three model norm combinations: **(A)** [1,2,2,2], **(B)** [1,1,1,1], **(C)** [0,2,2,2] and histogram showing the distribution of residuals. Orange line is the median of the residuals, green line is the mean, and blue line is the kernel density estimate (KDE). Root Mean Square Error (RMSE) of the residuals shown for each histogram.

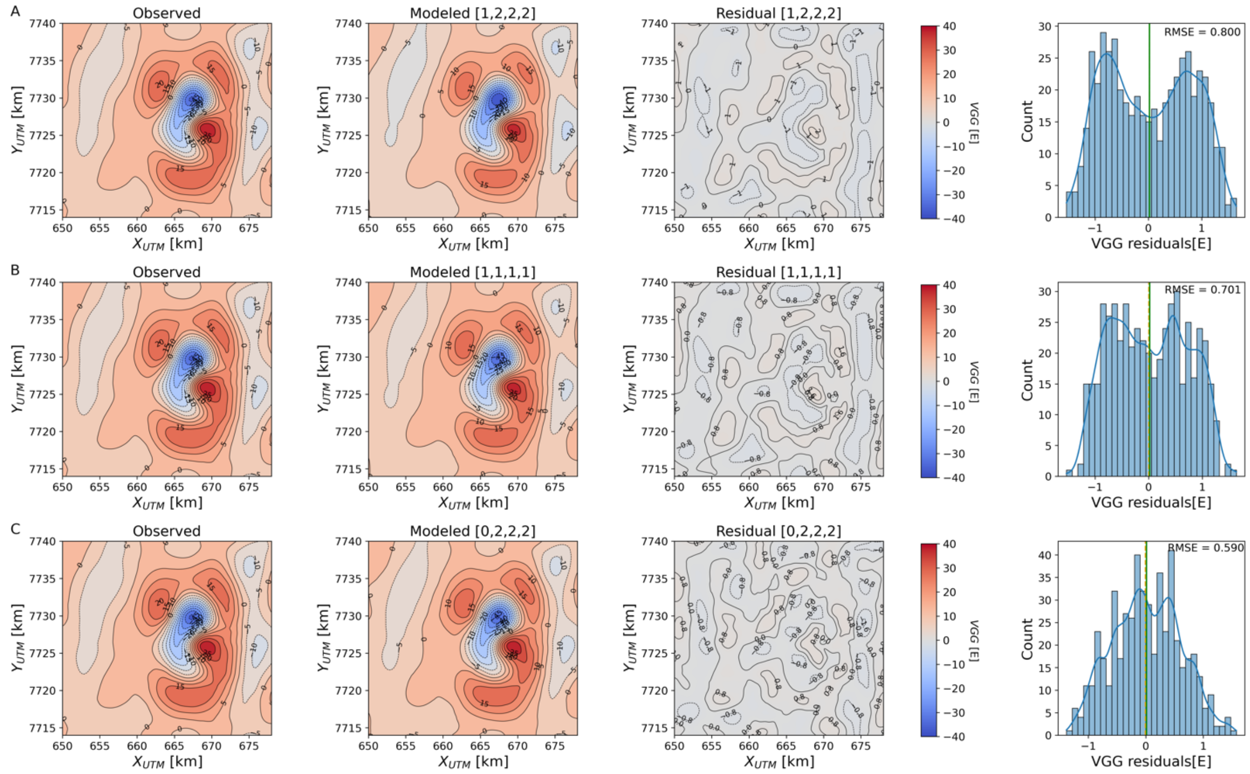

Fig. S20. Model misfit for the co-eruptive VGG Bouguer anomaly. Observed VGG anomaly, modeled, and residuals for three model norm combinations: **(A)** [1,2,2,2], **(B)** [1,1,1,1], **(C)** [0,2,2,2] and histogram showing the distribution of residuals. Orange line is the median of the residuals, green line is the mean, and blue line is the kernel density estimate (KDE). Root Mean Square Error (RMSE) of the residuals shown for each histogram.

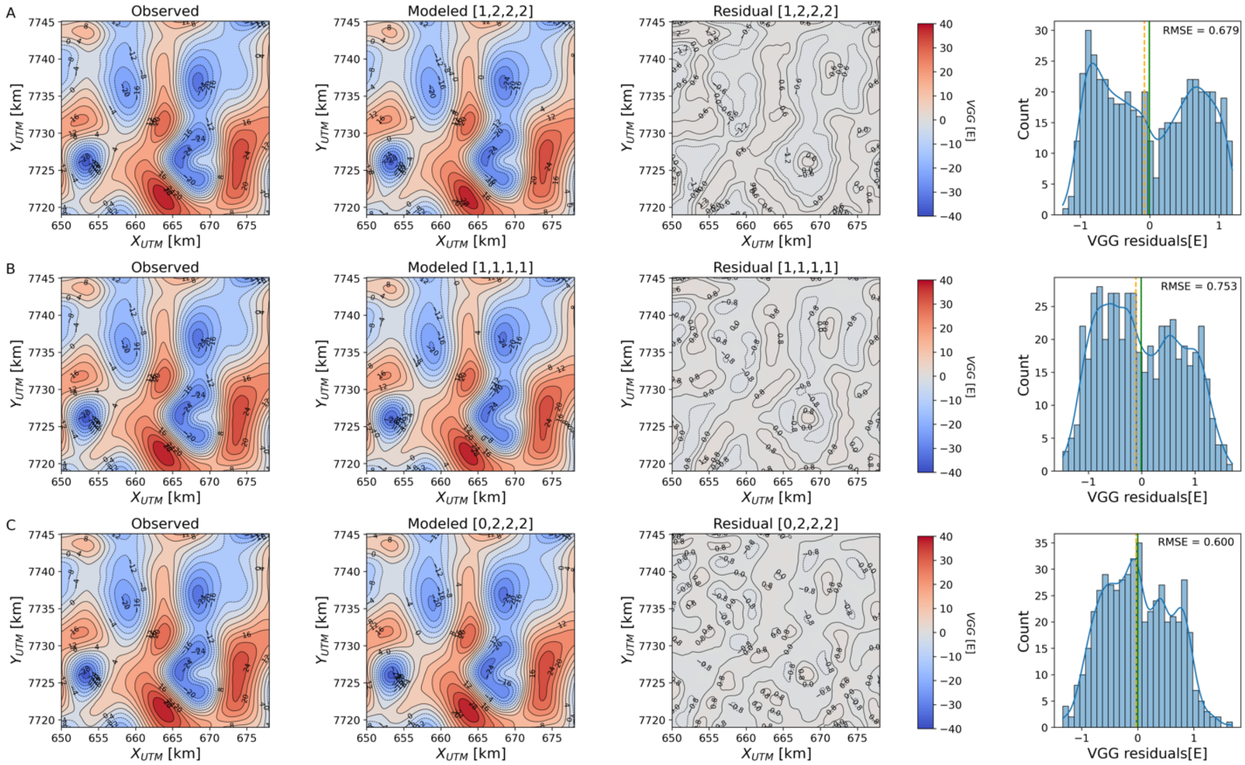

Fig. S21. Model misfit for the post-eruptive VGG Bouguer anomaly. Observed VGG anomaly, modeled, and residuals for three model norm combinations: **(A)** [1,2,2,2], **(B)** [1,1,1,1], **(C)** [0,2,2,2] and histogram showing the distribution of residuals. Orange line is the median of the residuals, green line is the mean, and blue line is the kernel density estimate (KDE). Root Mean Square Error (RMSE) of the residuals shown for each histogram.

Captions for the supplementary data files

Data S1. Density model. Archive contains three .csv files of the pre-, co-, and post-eruptive density model values as displayed on Figure 7. X, Y (UTM coordinates), and Z (depth) in meters and density contrast in the fourth column in g/cm^3.

Data S2. Post-eruptive DEM. XYZ file (longitude and latitude in decimal degrees, elevation in meters) of the post-eruptive DEM with 3 arcsecond resolution (~90 m), shown on Figure 2H.
